# Author Correction: Genetic variants in UNC93B1 predispose to childhood-onset systemic lupus erythematosus

**DOI:** 10.1038/s41590-024-01969-9

**Published:** 2024-09-09

**Authors:** Mahmoud Al-Azab, Elina Idiiatullina, Ziyang Liu, Meng Lin, Katja Hrovat-Schaale, Huifang Xian, Jianheng Zhu, Mandy Yang, Bingtai Lu, Zhiyao Zhao, Yiyi Liu, Jingjie Chang, Xiaotian Li, Caiqin Guo, Yunfeng Liu, Qi Wu, Jiazhang Chen, Chaoting Lan, Ping Zeng, Jun Cui, Xia Gao, Wenhao Zhou, Yan Zhang, Yuxia Zhang, Seth L. Masters

**Affiliations:** 1 Department of Immunology, Guangzhou Institute of Paediatrics, Guangzhou Women and Children’s Medical Centre, and State Key Laboratory of Respiratory Diseases, Guangzhou Medical University, Guangzhou, China; 2 Department of Medical Microbiology, Faculty of Medicine, University of Science and Technology, Aden, Yemen; 3 Department of Therapy and Nursing, Bashkir State Medical University, Ufa, Russia; 4 Inflammation Division, Walter and Eliza Hall Institute of Medical Research, Parkville, Victoria Australia; 5 Department of Medical Biology, University of Melbourne, Parkville, Victoria Australia; 6 State Key Laboratory of Respiratory Diseases, School of Basic Medical Sciences, Guangzhou Medical University, Guangzhou, China; 7 Center for Mitochondrial Genetics and Health, Greater Bay Area Institute of Precision Medicine (Guangzhou), Guangzhou, China; 8 Clinical Laboratory, Guangzhou Women and Children’s Medical Centre, Guangzhou Medical University, Guangdong, China; 9 National Children Medical Center, Department of Clinical Immunology, Children’s Hospital of Fudan University, Shanghai, China; 10 School of Life Sciences, Sun Yat-sen University, Guangzhou, China; 11 Centre for Innate Immunity and Infectious Diseases, Hudson Institute of Medical Research, Clayton, Victoria Australia; 12 Department of Molecular and Translational Science, Monash University, Clayton, Victoria Australia

**Keywords:** Toll-like receptors, Systemic lupus erythematosus

Correction to: *Nature Immunology* 10.1038/s41590-024-01846-5, published online 3 June 2024.

In the version of the article initially published, two references were missing which have now been added to the HTML and PDF versions of the article as refs. 36 and 37: Wolf, C. et al. UNC93B1 variants underlie TLR7-dependent autoimmunity. *Sci. Immunol*. **9**, eadi9769 (2024) and Mishra, H. et al. Disrupted degradative sorting of TLR7 is associated with human lupus. *Sci. Immunol*. **9**, eadi9575 (2024).

