# Supplementary material for: Genetic variants in UNC93B1 predispose to childhood-onset systemic lupus erythematosus
Source: Nat Immunol. 2024 Jun 3;25(6):969–80. doi: 10.1038/s41590-024-01846-5 (PMC11147776; doi:10.1038/s41590-024-01846-5)
Supplement: Supplementary file 1 — Supplementary Tables 1–4 and Figs. 1 and 2. [file 41590_2024_1846_MOESM1_ESM.pdf]

nature immunology

Supplementary information https://doi.org/10.1038/s41590-024-01846-5

Genetic variants in UNC93B1 predispose to childhood-onset systemic lupus erythematosus

In the format provided by the authors and unedited

Supplementary Table 1

Incidence of UNC93B1 (V117L) in South Coast Han with and without childhood-onset SLE

Genotype	V117L	WT	Total	Chi-square	p-value	Odds Ratio
Childhood-onset SLE	7	265	272			
South Coast Han	6	4057	4063	50.18	0.000004391946488	17.9
Total	13	4322	4335			

Contingency analysis, p value from Fishers exact test (Two-sided), odds ratio from Baptista-Pike test, Chi-square from Chi-square test. OR 95 % CI: 6.54 to 53.5

Supplementary Table 2

Genetic characteristics of patients with UNC93B1 variants

	Patient 1 (T314A)	Patient 2-8 (V117L)
POS	11:67996751	11:68003065
REF/ALT	T/C	C/A
SNP	NA	rs772328269
cDNA Change	c.A940G	c.G349T
Protein Change	p.T314A	p.V117L
LRT	Deleterious	Deleterious
MutationTaster	0.997 (Disease causing)	0.989 (Disease causing)
CADD	15.05	16.29

Supplementary Table 3

Clinical parameters for patients with UNC93B1 variants (P8 was unavailable).

**Clinical presentation**	**P1 (T314A)**	**P2 (V117L)**	**P3 (V117L)**	**P4 (V117L)**	**P5 (V117L)**	**P6 (V117L)**	**P7 (V117L)**
Age of diagnosis (years)	8	9	8	10	10	12	13
Fatigue	-	-	+	-	+	-	-
Fever	+	-	+	+	-	+	+
Alopecia	-	+	+	-	-	-	-
Arthritis and arthralgias	-	-	-	-	-	+	+
Mucocutaneous involvement	+	+	+	+	+	+	+
Photosensitivity	-	-	-	-	-	-	+
Cardiac involvement	-	-	+	+	-	-	-
Vascular manifestations	-	-	-	+	-	-	-
Renal involvement	+	+	+	+	-	+	-
Gastrointestinal involvement	-	+	+	+	-	-	+
Pulmonary involvement	-	-	+	+	+	-	-
Hematologic abnormalities	+	-	+	+	-	-	+
Ophthalmologic involvement	+	-	-	-	-	-	+
**Laboratory tests**							
Anti-Sm	-	-	-	+	-	-	-
Anti-Ro/SSA	+	+	+	-	-	+	+
Anti-La/SSB	-	-	-	-	-	-	-
Anti-nRNP	-	+	-	+	-	-	-
Coomb's test	No data	-	++	No data	+++	+	++++
Hematuria	+++	++++	+++	+++	-	-	+
Proteinuria	++	++++	++++	++	+	-	-
Urine WBC	-	+	+	++	++	+	+
**Therapy (chronological)**							
	MSS	OS	BED	MSS	PS	PS	MSS
	OS	MSS	TM	OS	MSS	MM	CCP
	PS	OH	VD	CCP	HS	HS	GG
	MM	CG	CG	GG	CCP	CCP	D-CAL
	VD	HS	PS	LT	GG	D-CAL	PC
	CG	VD	MM	PS	D-CAL	CA	OS
	CF	GG		VD	MM	MSS	GRL
	GSG	THY		MTX	CA		BED
	FS	CPP		FA			CA
	DD	MM					CYC
	CPP	BM					
	GRL	DS					
	LT	BFG					
		GOG					

MSS, methylprednisolone sodium succinate; OS, omeprazole sodium; CPP, cyclophosphamide; GG, glutamine granules; LT, loratadine; PS, prednisone; MTX, methotrexate; FA, folic acid; OH, ondansetron hydrochloride; CG, compound glycyrrhizin; HS, hydroxychloroquine sulfate; THY, thymosin; BM, Belimumab; DS, dexamethasone

sodium; BFG, bovine basic fibroblast growth factor eye gel; GOG, Ganciclovir Ophthalmic Gel; BED, brinzolamide eye drops; TM, timolol maleate eye drops; CF, compound falcodine; MM, mycophenolate mofetil; VD, Vitamin D; GSG, L-glutamine sodium gualenate; FS, fosinopril sodium; DD, Dipyridamole; GRL, Glucuronolactone, PC, potassium citrate; CA, Calcitriol; D-CAL, calcium carbonate and vitamin D3; CYC, Cyclosporin.

Supplementary Table 4

Primers used for QPCR

Gene	Species	Forward Primer	Reverse Primer
IFNB1	Human	AGTAGGCGACACTGTTCGTG	AGCCTCCCATTCAATTGCCA
IFIT3	Human	AAAAGCCCAACAACCCAGAAT	CGTATTGGTTATCAGGACTCAGC
ISG15	Human	CGCAGATCACCCAGAAGATCG	TTCGTCGCATTTGTCCACCA
ISG20L2	Human	AGGAAATGCCAAGCACCGAAA	TGAAGGTTCAGAGTGCAACTTAG
IL-8	Human	TTTTGCCAAGGAGTGCTAAAGA	AACCCTCTGCACCCAGTTTTC
IL12A	Human	CCTTGCACTTCTGAAGAGATTGA	ACAGGGCCATCATAAAAGAGGT
TNF	Human	CCTCTCTCTAATCAGCCCTCTG	GAGGACCTGGGAGTAGATGAG
MX1	Human	GTTTCCGAAGTGGACATCGCA	CTGCACAGGTTGTTCTCAGC
IFIT1	Human	TTGATGACGATGAAATGCCTGA	CAGGTCACCAGACTCCTCAC
GAPDH	Human	GGAGCGAGATCCCTCCAAAAT	GGCTGTTGTCATACTTCTCATGG
B2M	Human	AGCAGCATCATGGAGGTTTG	AGCCCTCCTAGAGCTACCTG
IL-6	Human	AGAGGCACTGGCAGAAAACAAC	AGGCAAGTCTCCTCATTGAATCC
ISG54	Human	AAGCACCTCAAAGGGCAAAAC	TCGGCCCATGTGATAGTAGAC
Irf-7	Mouse	AAGCTGGAGCCATGGGTATG	CGATGTCTTCGTAGAGACTGTTGG
Ifit1	Mouse	AGAGTCAAGGCAGGTTTCTG	TGTGAAGTGACATCTCAGCTG
Mx1	Mouse	GATCCGACTTCACTTCCAGATGG	CATCTCAGTGGTAGTCAACCC
Isg20l2	Mouse	ACCAACTTGGAGGCCTATGG	AGGGGTCAGCCAAGACAATG
Isg15	Mouse	CGATTTCCTGGTGTCCGTGA	AGCCAGAACTGGTCTTCGTG
Tnf	Mouse	CCAAATGGCCTCCCTCTCAT	TGGTGGTTTGCTACGACGTG
Gapdh	Mouse	ATCAAGAAGGTGGTGAAGCA	AGACAACCTGGTCCTCAGTGT

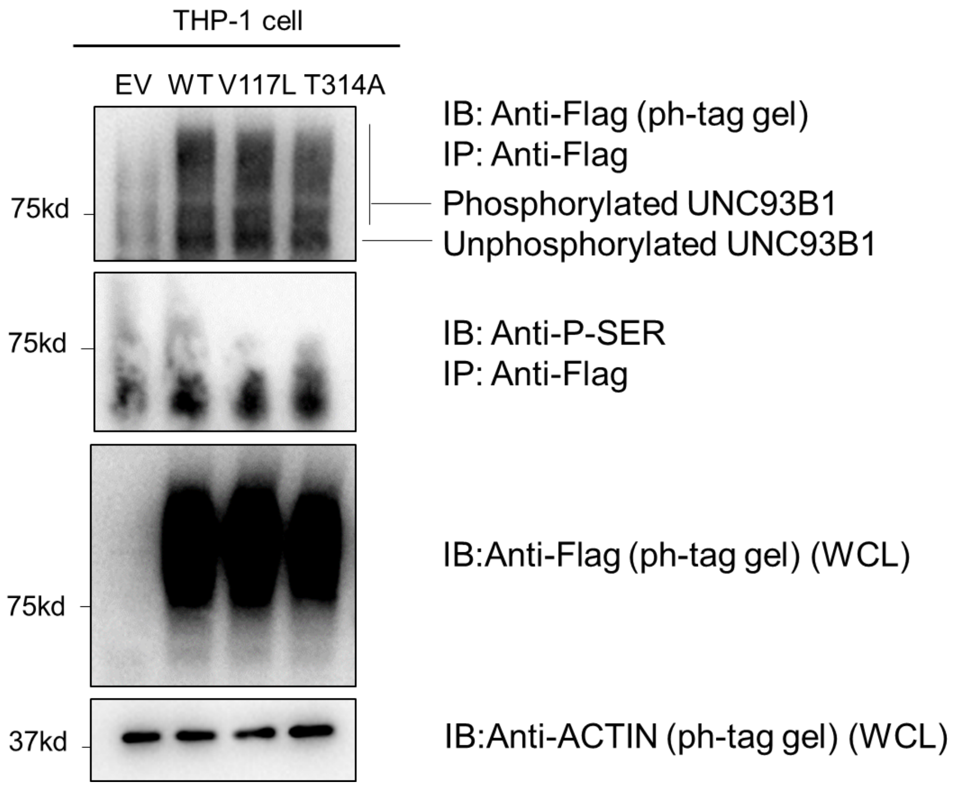

Supplementary Fig. 1| Immunoprecipitation and immunoblot of UNC93B1 phosphorylation using Ph-tag gel and P-ser antibody as indicated in THP-1 cells, n=3 biological replicates.

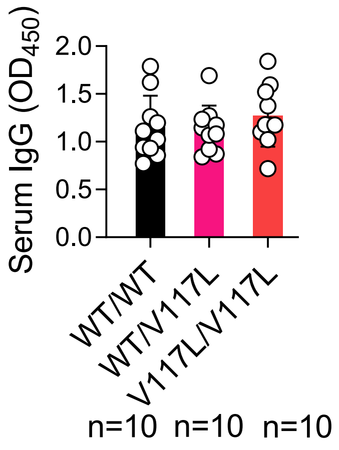

Supplementary Fig. 2| Serum IgG for indicated mice (n=10). Mice were age matched 8 months old, the data pooled from two independent experiments.

Source data of Supplementary Fig. 1

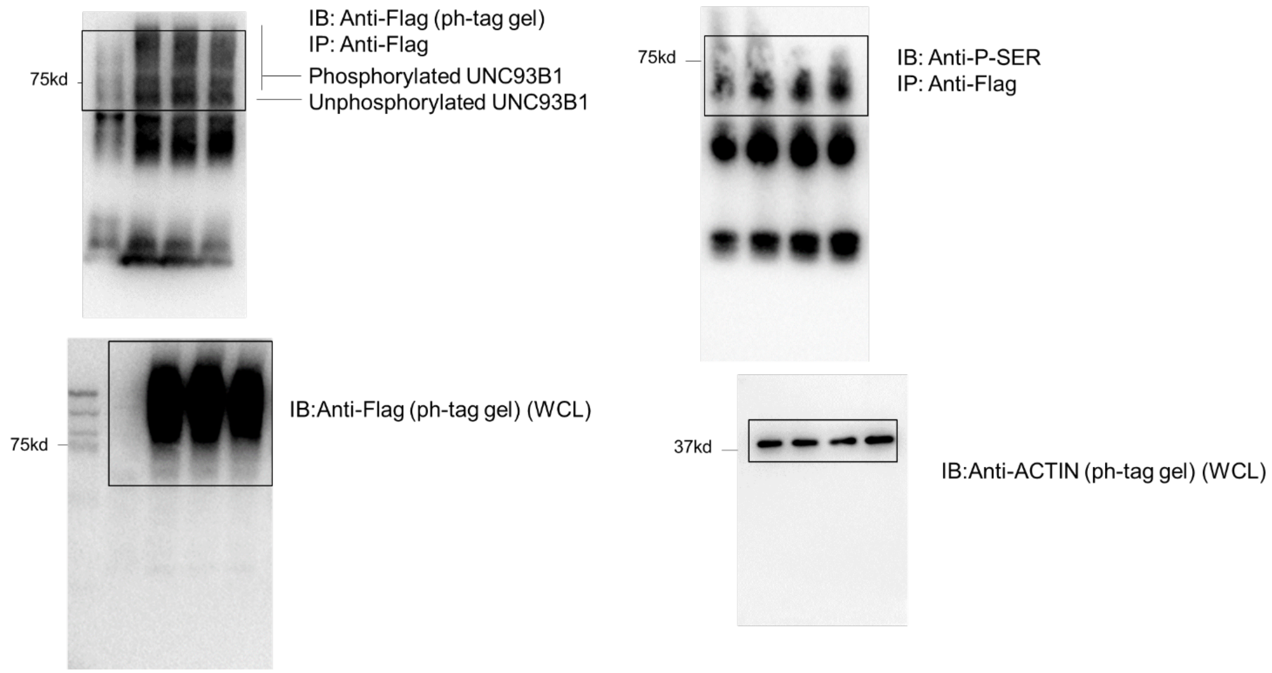

Source data of Supplementary Fig. 2

	WT/WT	WT/V117L	V117L/V117L
Serum IgG (OD450)	1.789	0.842	0.717
	1.026	0.873	1.374
	0.857	1.271	1.101
	1.262	0.923	1.176
	0.773	1.693	1.594
	1.112	1.146	1.521
	1.198	1.067	1.18
	1.622	1.235	1.176
	0.941	1.176	1.022
	0.93	1.082	1.842
