# Supplementary material for: Genetic variants in UNC93B1 predispose to childhood-onset systemic lupus erythematosus
Source: Nat Immunol. 2024 Jun 3;25(6):969–80. doi: 10.1038/s41590-024-01846-5 (PMC11147776; doi:10.1038/s41590-024-01846-5)
Supplement: Supplementary file 2 — Reporting Summary [file 41590_2024_1846_MOESM2_ESM.pdf]

nature portfolio

Corresponding author(s): Yuxia Zhang and Seth L. Masters

Last updated by author(s): Feb 21, 2024

Reporting Summary

Nature Portfolio wishes to improve the reproducibility of the work that we publish. This form provides structure for consistency and transparency in reporting. For further information on Nature Portfolio policies, see our Editorial Policies and the Editorial Policy Checklist.

Statistics

For all statistical analyses, confirm that the following items are present in the figure legend, table legend, main text, or Methods section.

n/a	Confirmed	
☐	☒	The exact sample size (*n*) for each experimental group/condition, given as a discrete number and unit of measurement
☐	☒	A statement on whether measurements were taken from distinct samples or whether the same sample was measured repeatedly
☐	☒	The statistical test(s) used AND whether they are one- or two-sided<br>*Only common tests should be described solely by name; describe more complex techniques in the Methods section.*
☐	☒	A description of all covariates tested
☐	☒	A description of any assumptions or corrections, such as tests of normality and adjustment for multiple comparisons
☐	☒	A full description of the statistical parameters including central tendency (e.g. means) or other basic estimates (e.g. regression coefficient) AND variation (e.g. standard deviation) or associated estimates of uncertainty (e.g. confidence intervals)
☐	☒	For null hypothesis testing, the test statistic (e.g. *F*, *t*, *r*) with confidence intervals, effect sizes, degrees of freedom and *P* value noted<br>*Give P values as exact values whenever suitable.*
☒	☐	For Bayesian analysis, information on the choice of priors and Markov chain Monte Carlo settings
☒	☐	For hierarchical and complex designs, identification of the appropriate level for tests and full reporting of outcomes
☒	☐	Estimates of effect sizes (e.g. Cohen's *d*, Pearson's *r*), indicating how they were calculated

Our web collection on statistics for biologists contains articles on many of the points above.

Software and code

Policy information about availability of computer code

Data collection	No special software was used.
Data analysis	The sequencing reads for human THP-1 cells were then aligned to human reference genome GRCh38 using STAR aligner (ver. 2.7.9a) 37 to generate the binary alignment file (.bam), which was then passed to HTSeq for gene expression quantification using the htseq-count function (ver. 0.11.1) 38. The resulting gene expression matrix was then normalized and differentially expressed genes (DEGs) were identified using R package DESeq2 (ver. 1.34.0) 39. Genes with adjusted p value less than 0.05 and absolute fold change (log2 space) > 1 were considered as DEGs. This analysis pipeline was used for the volcano plots in Figure 3. Other analysis was performed using BGI software (https://biosys.bgi.com/#/report/login). CYTEK NL-CLC software, SpectroFlo and FlwoJo_v10.8.1 used for analysis of flow cytometry data. Developed membranes of immunoblotting were imaged using the Image Lab detection system (Bio-Rad, USA). Structural analysis software, Pymol V2.5.8. Statistical analysis was performed using Prism 9 software (GraphPad Software Inc.)

For manuscripts utilizing custom algorithms or software that are central to the research but not yet described in published literature, software must be made available to editors and reviewers. We strongly encourage code deposition in a community repository (e.g. GitHub). See the Nature Portfolio guidelines for submitting code & software for further information.

Data

Policy information about availability of data

All manuscripts must include a data availability statement. This statement should provide the following information, where applicable:

- Accession codes, unique identifiers, or web links for publicly available datasets
- A description of any restrictions on data availability
- For clinical datasets or third party data, please ensure that the statement adheres to our policy

The RNA-seq raw sequencing data for THP-1 cell line reported in this study are deposited under supervision and control of the Genome Sequence Archive of the Beijing Institute of Genomics (BIG), Chinese Academy of Sciences (https://ngdc.cncb.ac.cn/gsa-human/), under accession number HRA005592. THP-1 RNA-seq data of this project could be accessed after an approval application. Please refer to GSA (https://ngdc.cncb.ac.cn/gsa-human/) for detailed application guidance. The RNA-seq raw sequencing data for mouse model reported in this study are deposited under supervision and control of the Genome Sequence Archive of BIG), Chinese Academy of Sciences (https://ngdc.cncb.ac.cn/gsa/), under accession number CRA012690. For access to mouse RNA-seq data of this project, please contact the corresponding authors for approval. All other data including raw immunoblot and fluorescence image files, and other in vitro experimental data used to support the findings of this study available in the main text or the supplementary materials.

Research involving human participants, their data, or biological material

Policy information about studies with human participants or human data. See also policy information about sex, gender (identity/presentation), and sexual orientation and race, ethnicity and racism.

Reporting on sex and gender	All 8 patients were female.
Reporting on race, ethnicity, or other socially relevant groupings	We found that the gene variant of interest was present in the East Asian population, and is most prevalent in South Coast Han.
Population characteristics	Female patients (P) 1-8 at age of 8-26 years respectively presented symptoms of systemic inflammation associated with lupus nephritis. All healthy controls were females with age matched to P2 and P3 age.
Recruitment	We retrieved the peripheral blood samples of patients with SLE and healthy control individuals from Clinical Biological Resource Bank of Guangzhou Women and Children's Medical Center. No potential self-selection bias was involved during the sample collection process.
Ethics oversight	Written informed consent has been obtained from the authorized individual for all participated patients. The Guangzhou Women and Children's Medical Centre Medical Ethics Committee approved the study procedures ([2021]073B00) in consistence with Helsinki Declaration about ethics in using human samples.

Note that full information on the approval of the study protocol must also be provided in the manuscript.

Field-specific reporting

Please select the one below that is the best fit for your research. If you are not sure, read the appropriate sections before making your selection.

☒ Life sciences ☐ Behavioural & social sciences ☐ Ecological, evolutionary & environmental sciences

For a reference copy of the document with all sections, see nature.com/documents/nr-reporting-summary-flat.pdf

Life sciences study design

All studies must disclose on these points even when the disclosure is negative.

Sample size	For mouse experiments, at least 3 per group were studied, and more if required to achieve statistical significance. No statistical methods were used to pre-determine sample sizes but our sample sizes are similar to those reported in previous publications.
Data exclusions	No data were excluded from the analyses unless technically detected errors.
Replication	The experiments were performed by at least three biological replicates. The details were described in the Figure Legends and Methods.
Randomization	For animal studies, randomization was performed by the animal facility.
Blinding	Blinding was applied for disease scoring of tissues.

Behavioural & social sciences study design

All studies must disclose on these points even when the disclosure is negative.

Study description	*Briefly describe the study type including whether data are quantitative, qualitative, or mixed-methods (e.g. qualitative cross-sectional, quantitative experimental, mixed-methods case study).*
Research sample	*State the research sample (e.g. Harvard university undergraduates, villagers in rural India) and provide relevant demographic information (e.g. age, sex) and indicate whether the sample is representative. Provide a rationale for the study sample chosen. For studies involving existing datasets, please describe the dataset and source.*
Sampling strategy	*Describe the sampling procedure (e.g. random, snowball, stratified, convenience). Describe the statistical methods that were used to predetermine sample size OR if no sample-size calculation was performed, describe how sample sizes were chosen and provide a rationale for why these sample sizes are sufficient. For qualitative data, please indicate whether data saturation was considered, and what criteria were used to decide that no further sampling was needed.*
Data collection	*Provide details about the data collection procedure, including the instruments or devices used to record the data (e.g. pen and paper, computer, eye tracker, video or audio equipment) whether anyone was present besides the participant(s) and the researcher, and whether the researcher was blind to experimental condition and/or the study hypothesis during data collection.*
Timing	*Indicate the start and stop dates of data collection. If there is a gap between collection periods, state the dates for each sample cohort.*
Data exclusions	*If no data were excluded from the analyses, state so OR if data were excluded, provide the exact number of exclusions and the rationale behind them, indicating whether exclusion criteria were pre-established.*
Non-participation	*State how many participants dropped out/declined participation and the reason(s) given OR provide response rate OR state that no participants dropped out/declined participation.*
Randomization	*If participants were not allocated into experimental groups, state so OR describe how participants were allocated to groups, and if allocation was not random, describe how covariates were controlled.*

Ecological, evolutionary & environmental sciences study design

All studies must disclose on these points even when the disclosure is negative.

Study description	*Briefly describe the study. For quantitative data include treatment factors and interactions, design structure (e.g. factorial, nested, hierarchical), nature and number of experimental units and replicates.*
Research sample	*Describe the research sample (e.g. a group of tagged Passer domesticus, all Stenocereus thurberi within Organ Pipe Cactus National Monument), and provide a rationale for the sample choice. When relevant, describe the organism taxa, source, sex, age range and any manipulations. State what population the sample is meant to represent when applicable. For studies involving existing datasets, describe the data and its source.*
Sampling strategy	*Note the sampling procedure. Describe the statistical methods that were used to predetermine sample size OR if no sample-size calculation was performed, describe how sample sizes were chosen and provide a rationale for why these sample sizes are sufficient.*
Data collection	*Describe the data collection procedure, including who recorded the data and how.*
Timing and spatial scale	*Indicate the start and stop dates of data collection, noting the frequency and periodicity of sampling and providing a rationale for these choices. If there is a gap between collection periods, state the dates for each sample cohort. Specify the spatial scale from which the data are taken*
Data exclusions	*If no data were excluded from the analyses, state so OR if data were excluded, describe the exclusions and the rationale behind them, indicating whether exclusion criteria were pre-established.*
Reproducibility	*Describe the measures taken to verify the reproducibility of experimental findings. For each experiment, note whether any attempts to repeat the experiment failed OR state that all attempts to repeat the experiment were successful.*
Randomization	*Describe how samples/organisms/participants were allocated into groups. If allocation was not random, describe how covariates were controlled. If this is not relevant to your study, explain why.*
Blinding	*Describe the extent of blinding used during data acquisition and analysis. If blinding was not possible, describe why OR explain why blinding was not relevant to your study.*

Did the study involve field work? ☐ Yes ☒ No

Field work, collection and transport

Field conditions	*Describe the study conditions for field work, providing relevant parameters (e.g. temperature, rainfall).*
Location	*State the location of the sampling or experiment, providing relevant parameters (e.g. latitude and longitude, elevation, water depth).*
Access & import/export	*Describe the efforts you have made to access habitats and to collect and import/export your samples in a responsible manner and in compliance with local, national and international laws, noting any permits that were obtained (give the name of the issuing authority, the date of issue, and any identifying information).*
Disturbance	*Describe any disturbance caused by the study and how it was minimized.*

Reporting for specific materials, systems and methods

We require information from authors about some types of materials, experimental systems and methods used in many studies. Here, indicate whether each material, system or method listed is relevant to your study. If you are not sure if a list item applies to your research, read the appropriate section before selecting a response.

Materials & experimental systems

n/a	Involved in the study	
☐	☒	Antibodies
☐	☒	Eukaryotic cell lines
☒	☐	Palaeontology and archaeology
☐	☒	Animals and other organisms
☒	☐	Clinical data
☒	☐	Dual use research of concern
☒	☐	Plants

Methods

n/a	Involved in the study	
☒	☐	ChIP-seq
☐	☒	Flow cytometry
☒	☐	MRI-based neuroimaging

Antibodies

Antibodies used

For immunoblots the following antibodies were used: anti-FLAG (F1804, Sigma-Aldrich), anti-NF-κB p65 (8242S, Cell Signaling), anti-Phospho-NF-κB p65 (Ser468) (3039S, Cell Signaling), anti-JNK123 (ab179461, Abcam), anti- Phospho-JNK123 (ab124956, Abcam), anti-P38 (#8690 , Cell Signaling), anti-Phospho-P38 (9211S, Cell Signaling), anti-IRF5 (#20261, Cell Signaling), anti-Phospho- IRF5 (Ser437) (#PA5-64760, Invitrogen), anti-IRAK4 (#4363, Cell Signaling), anti- Phospho-IRAK4 (Thr345/Ser346) (#11927, Cell Signaling), anti-p44/42 MAPK (ERK1/2) (9102S, Cell Signaling), anti- Phospho-p44/42 MAPK (ERK1/2) (Thr202/Tyr204) (9101S, Cell Signaling), anti-IRF7 (#39659, Cell Signaling), anti-Phospho- IRF7 (Ser437/438) (#24129, Cell Signaling), anti-p-Ser/Phosphoserine (sc-81514, Santa Cruz Biotechnology), anti-β-actin (AC026, ABclonal), anti-rabbit IgG, HRP-linked antibody (7074, Cell Signaling), and anti-mouse IgG, HRP-linked antibody (7076, Cell Signaling). The dilution of anti-β-actin, anti-rabbit IgG HRP-linked antibody, anti-mouse IgG HRP-linked antibody, and anti-FLAG was 1:5000. The dilution of anti-p-Ser/Phosphoserine was 1:1000. The dilution for other primary antibodies was 1:1500.

For flow cytometry the following antibodies were used: anti-mouse TNF-α (506324, 1:200, Biolegend), anti-mouse IFN-γ (505830, 1:400, Biolegend), anti-mouse CD45 (103132, 1:200, Biolegend), anti-mouse F4/80 (123137, 1:200, Biolegend), anti-mouse/human CD11b (101205, 1:500, Biolegend), anti-mouse CD14 (123335, 1:200, Biolegend), anti-mouse CD16 (158004, 1:100, Biolegend), anti-mouse CD3 (100248, 1:100 Biolegend), anti-mouse CD4 (100406, 1:400, Biolegend), anti-mouse FOXP3 (126407, 1:200, Biolegend), anti-mouse CD25 (102043, 1:300, Biolegend), anti-mouse/human CD44 (103059, 1:100, Biolegend), anti-mouse CD69 (104507 or 104510, 1:200 , Biolegend), anti-mouse Gr-1 (108416, 1:200, Biolegend), anti-mouse CD19 (159812, 1:400, Biolegend), anti-mouse CD95 (152612, 1:200, Biolegend), anti- mouse/human Bcl-6 (358510, 1:100, Biolegend), anti- mouse/human CD45R/B220 (103222, 1:200, , Biolegend), and anti-mouse CXCR5 (145517, 1:100, Biolegend).

Validation

All antibodies used in our study are commercial and any validation statements are noted on manufacture's website.

Eukaryotic cell lines

Policy information about cell lines and Sex and Gender in Research

Cell line source(s)	THP-1 cells were obtained from American Type Culture Collection (ATCC) and cultured accordingly.
Authentication	The authentication procedures for cell line were done by the supplier.
Mycoplasma contamination	All cells used in our study were confirmed that had no Mycoplasma contamination.

Commonly misidentified lines (See ICLAC register)	No misidentified cell lines were used in our study.

Palaeontology and Archaeology

Specimen provenance	*Provide provenance information for specimens and describe permits that were obtained for the work (including the name of the issuing authority, the date of issue, and any identifying information). Permits should encompass collection and, where applicable, export.*
Specimen deposition	*Indicate where the specimens have been deposited to permit free access by other researchers.*
Dating methods	*If new dates are provided, describe how they were obtained (e.g. collection, storage, sample pretreatment and measurement), where they were obtained (i.e. lab name), the calibration program and the protocol for quality assurance OR state that no new dates are provided.*

☐ Tick this box to confirm that the raw and calibrated dates are available in the paper or in Supplementary Information.

Ethics oversight	*Identify the organization(s) that approved or provided guidance on the study protocol, OR state that no ethical approval or guidance was required and explain why not.*

Note that full information on the approval of the study protocol must also be provided in the manuscript.

Animals and other research organisms

Policy information about studies involving animals; ARRIVE guidelines recommended for reporting animal research, and Sex and Gender in Research

Laboratory animals	C57BL/6J mice lines (UNC93B1WT/WT, UNC93B1WT/V117L, and UNC93B1V117L/V117L). Mice were age matched 8-14 months old.
Wild animals	No wild animals were used in the study.
Reporting on sex	All mice used for analysis in this project are females.
Field-collected samples	No field collected samples were used in the study.
Ethics oversight	Animal studies were approved by Institutional Animal Care and Use Committee of Guangzhou Medical University (GY2022-035)

Note that full information on the approval of the study protocol must also be provided in the manuscript.

Clinical data

Policy information about clinical studies

All manuscripts should comply with the ICMJE guidelines for publication of clinical research and a completed CONSORT checklist must be included with all submissions.

Clinical trial registration	*Provide the trial registration number from ClinicalTrials.gov or an equivalent agency.*
Study protocol	*Note where the full trial protocol can be accessed OR if not available, explain why.*
Data collection	*Describe the settings and locales of data collection, noting the time periods of recruitment and data collection.*
Outcomes	*Describe how you pre-defined primary and secondary outcome measures and how you assessed these measures.*

Dual use research of concern

Policy information about dual use research of concern

Hazards

Could the accidental, deliberate or reckless misuse of agents or technologies generated in the work, or the application of information presented in the manuscript, pose a threat to:

No	Yes	
☐	☐	Public health
☐	☐	National security
☐	☐	Crops and/or livestock
☐	☐	Ecosystems
☐	☐	Any other significant area

Experiments of concern

Does the work involve any of these experiments of concern:

No	Yes	
☐	☐	Demonstrate how to render a vaccine ineffective
☐	☐	Confer resistance to therapeutically useful antibiotics or antiviral agents
☐	☐	Enhance the virulence of a pathogen or render a nonpathogen virulent
☐	☐	Increase transmissibility of a pathogen
☐	☐	Alter the host range of a pathogen
☐	☐	Enable evasion of diagnostic/detection modalities
☐	☐	Enable the weaponization of a biological agent or toxin
☐	☐	Any other potentially harmful combination of experiments and agents

Plants

Seed stocks	*Report on the source of all seed stocks or other plant material used. If applicable, state the seed stock centre and catalogue number. If plant specimens were collected from the field, describe the collection location, date and sampling procedures.*
Novel plant genotypes	*Describe the methods by which all novel plant genotypes were produced. This includes those generated by transgenic approaches, gene editing, chemical/radiation-based mutagenesis and hybridization. For transgenic lines, describe the transformation method, the number of independent lines analyzed and the generation upon which experiments were performed. For gene-edited lines, describe the editor used, the endogenous sequence targeted for editing, the targeting guide RNA sequence (if applicable) and how the editor was applied.*
Authentication	*Describe any authentication procedures for each seed stock used or novel genotype generated. Describe any experiments used to assess the effect of a mutation and, where applicable, how potential secondary effects (e.g. second site T-DNA insertions, mosiacism, off-target gene editing) were examined.*

ChIP-seq

Data deposition

☐ Confirm that both raw and final processed data have been deposited in a public database such as GEO.

☐ Confirm that you have deposited or provided access to graph files (e.g. BED files) for the called peaks.

Data access links<br>*May remain private before publication.*	*For "Initial submission" or "Revised version" documents, provide reviewer access links. For your "Final submission" document, provide a link to the deposited data.*
Files in database submission	*Provide a list of all files available in the database submission.*
Genome browser session<br>(e.g. UCSC)	*Provide a link to an anonymized genome browser session for "Initial submission" and "Revised version" documents only, to enable peer review. Write "no longer applicable" for "Final submission" documents.*

Methodology

Replicates	*Describe the experimental replicates, specifying number, type and replicate agreement.*
Sequencing depth	*Describe the sequencing depth for each experiment, providing the total number of reads, uniquely mapped reads, length of reads and whether they were paired- or single-end.*
Antibodies	*Describe the antibodies used for the ChIP-seq experiments; as applicable, provide supplier name, catalog number, clone name, and lot number.*
Peak calling parameters	*Specify the command line program and parameters used for read mapping and peak calling, including the ChIP, control and index files used.*

Data quality | *Describe the methods used to ensure data quality in full detail, including how many peaks are at FDR 5% and above 5-fold enrichment.*

Software | *Describe the software used to collect and analyze the ChIP-seq data. For custom code that has been deposited into a community repository, provide accession details.*

Flow Cytometry

Plots

Confirm that:

☒ The axis labels state the marker and fluorochrome used (e.g. CD4-FITC).

☒ The axis scales are clearly visible. Include numbers along axes only for bottom left plot of group (a 'group' is an analysis of identical markers).

☒ All plots are contour plots with outliers or pseudocolor plots.

☒ A numerical value for number of cells or percentage (with statistics) is provided.

Methodology

Sample preparation	The whole spleen was minced into tiny pieces in 6-well plate on ice using plunger end of the syringe and filtered by 70 µm strainer with rinsing by 5mL PBS with 2% FBS. The collected tissue filtrates were centrifuged at 500g /5 minutes at 4°C . The cells pallet suspended in RBC lysis buffer and incubated for 5 minutes at 25°C and then mixed with double volume of PBS with 2% FBS. The immune cells were collected in the pellet after centrifugation at 500g /5 minutes at 4°C .<br>Collected bone marrow from mouse femur bones by centrifuging using technique of two layers of tubes were incubated with RBC lysis buffer for 5 minutes at 25°C and then mixed with double volume of PBS with 2% FBS. The immune cells were collected in the pellet after centrifugation at 500g /5 minutes at 4°C.<br>For flow cytometry, 1 million cells were incubated with Zombie Aqua-A (Biolegend) for 15 min in dark, then washed to be stained by antibodies for cell surface markers and incubated for 30 min at 4°C and then washed. Fixation and permeabilization kit (88-8824-00, Thermofisher) was used for fixation and intracellular permeablization for intracellular and nuclear markers according to manufacturer's protocol.
Instrument	CYTEK NL-CLC
Software	SpectroFlo and FlwoJo_v10.8.1.
Cell population abundance	Gating strategies in the Extended Data Fig. 10
Gating strategy	The Extended Data Fig. 10 is the Representative gating strategies for myeloid cells in bone marrow, macrophage, monocytes, inflammatory Gr-1+ cells, and IFN-γ producing CD4 T cells and CD3+/CD4- cells, lymphocytes subsets, B cells, CD4 T cells, T regulatory cells, activated T cells, or/and germinal center B cells in bone marrow or/and spleen. These strategies used for data presented in figure 6.

☒ Tick this box to confirm that a figure exemplifying the gating strategy is provided in the Supplementary Information.

Magnetic resonance imaging

Experimental design

Design type	*Indicate task or resting state; event-related or block design.*
Design specifications	*Specify the number of blocks, trials or experimental units per session and/or subject, and specify the length of each trial or block (if trials are blocked) and interval between trials.*
Behavioral performance measures	*State number and/or type of variables recorded (e.g. correct button press, response time) and what statistics were used to establish that the subjects were performing the task as expected (e.g. mean, range, and/or standard deviation across subjects).*

Acquisition

Imaging type(s)	*Specify: functional, structural, diffusion, perfusion.*
Field strength	*Specify in Tesla*
Sequence & imaging parameters	*Specify the pulse sequence type (gradient echo, spin echo, etc.), imaging type (EPI, spiral, etc.), field of view, matrix size, slice thickness, orientation and TE/TR/flip angle.*
Area of acquisition	*State whether a whole brain scan was used OR define the area of acquisition, describing how the region was determined.*

Diffusion MRI ☐ Used ☐ Not used

Preprocessing

Preprocessing software — *Provide detail on software version and revision number and on specific parameters (model/functions, brain extraction, segmentation, smoothing kernel size, etc.).*

Normalization — *If data were normalized/standardized, describe the approach(es): specify linear or non-linear and define image types used for transformation OR indicate that data were not normalized and explain rationale for lack of normalization.*

Normalization template — *Describe the template used for normalization/transformation, specifying subject space or group standardized space (e.g. original Talairach, MNI305, ICBM152) OR indicate that the data were not normalized.*

Noise and artifact removal — *Describe your procedure(s) for artifact and structured noise removal, specifying motion parameters, tissue signals and physiological signals (heart rate, respiration).*

Volume censoring — *Define your software and/or method and criteria for volume censoring, and state the extent of such censoring.*

Statistical modeling & inference

Model type and settings — *Specify type (mass univariate, multivariate, RSA, predictive, etc.) and describe essential details of the model at the first and second levels (e.g. fixed, random or mixed effects; drift or auto-correlation).*

Effect(s) tested — *Define precise effect in terms of the task or stimulus conditions instead of psychological concepts and indicate whether ANOVA or factorial designs were used.*

Specify type of analysis: ☐ Whole brain ☐ ROI-based ☐ Both

Statistic type for inference — *Specify voxel-wise or cluster-wise and report all relevant parameters for cluster-wise methods.*

(See Eklund et al. 2016)

Correction — *Describe the type of correction and how it is obtained for multiple comparisons (e.g. FWE, FDR, permutation or Monte Carlo).*

Models & analysis

n/a	Involved in the study
☐	☐ Functional and/or effective connectivity
☐	☐ Graph analysis
☐	☐ Multivariate modeling or predictive analysis

Functional and/or effective connectivity — *Report the measures of dependence used and the model details (e.g. Pearson correlation, partial correlation, mutual information).*

Graph analysis — *Report the dependent variable and connectivity measure, specifying weighted graph or binarized graph, subject- or group-level, and the global and/or node summaries used (e.g. clustering coefficient, efficiency, etc.).*

Multivariate modeling and predictive analysis — *Specify independent variables, features extraction and dimension reduction, model, training and evaluation metrics.*
