# Supplementary figures and images for: Genetic variants in UNC93B1 predispose to childhood-onset systemic lupus erythematosus
Source: Nat Immunol. 2024 Jun 3;25(6):969–80. doi: 10.1038/s41590-024-01846-5 (PMC11147776; doi:10.1038/s41590-024-01846-5)

# Fig. 3b

p-NFκBp65

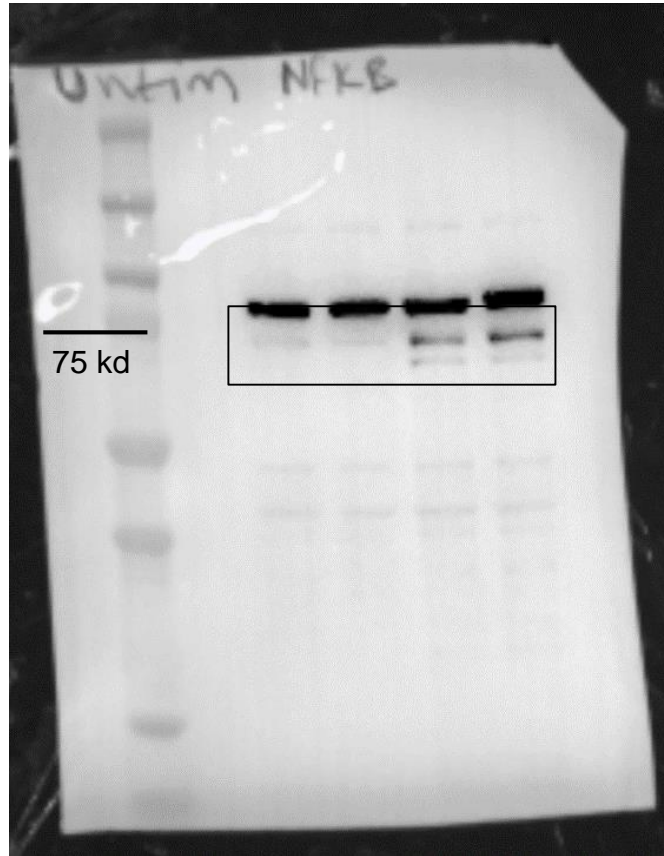

NFκBp65

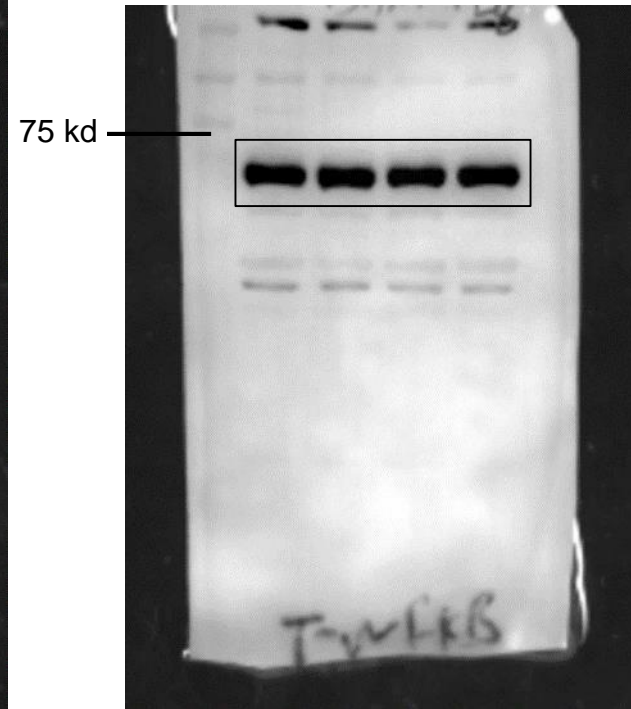

P-JNK123

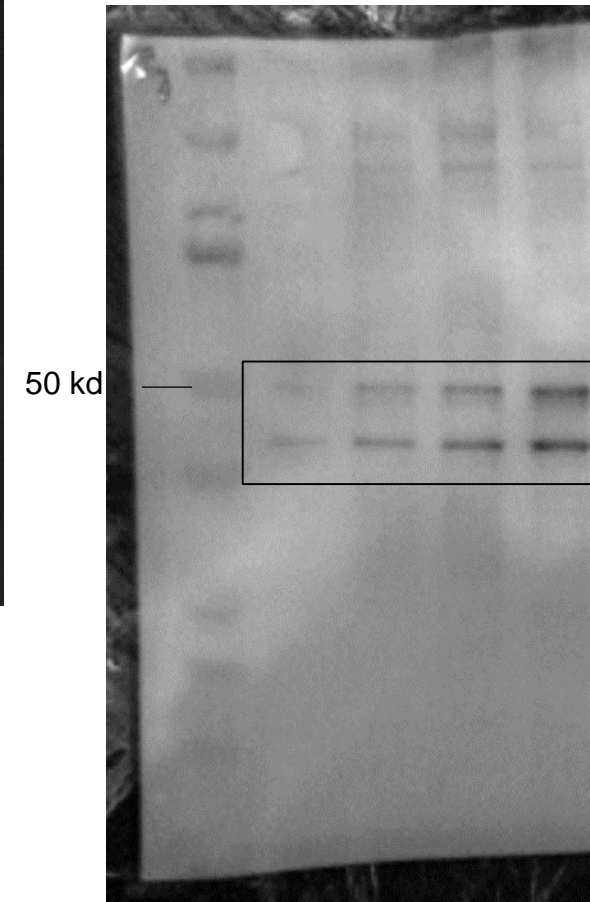

JNK123

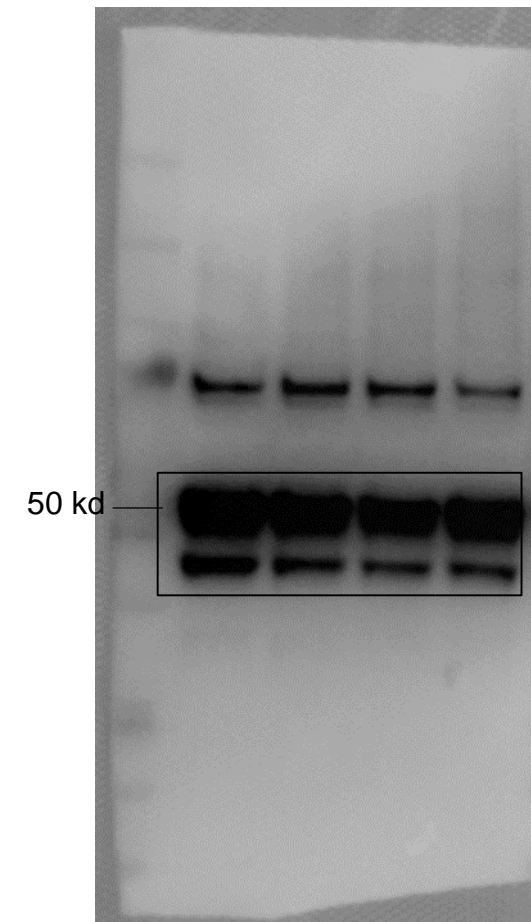

**Fig. 3b**

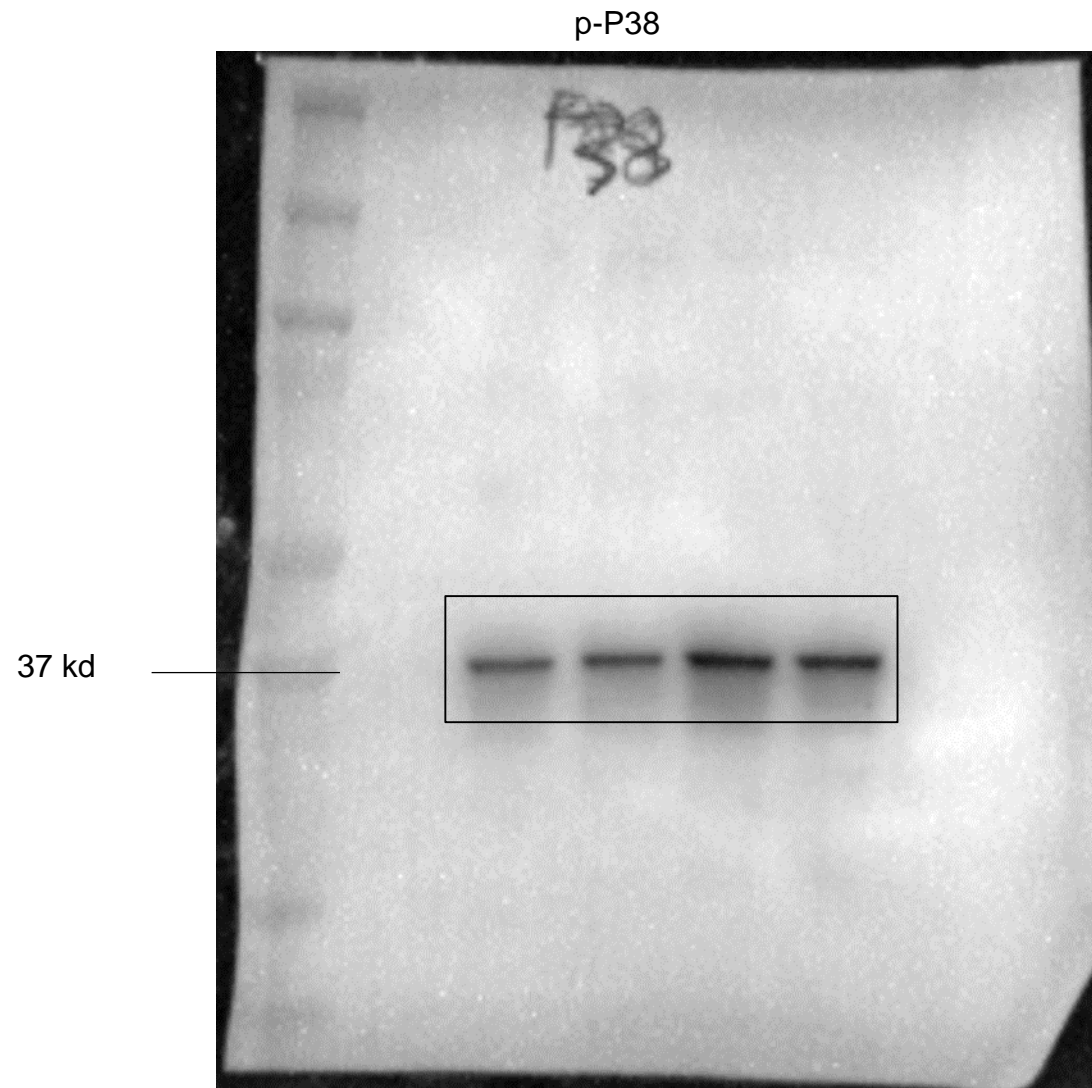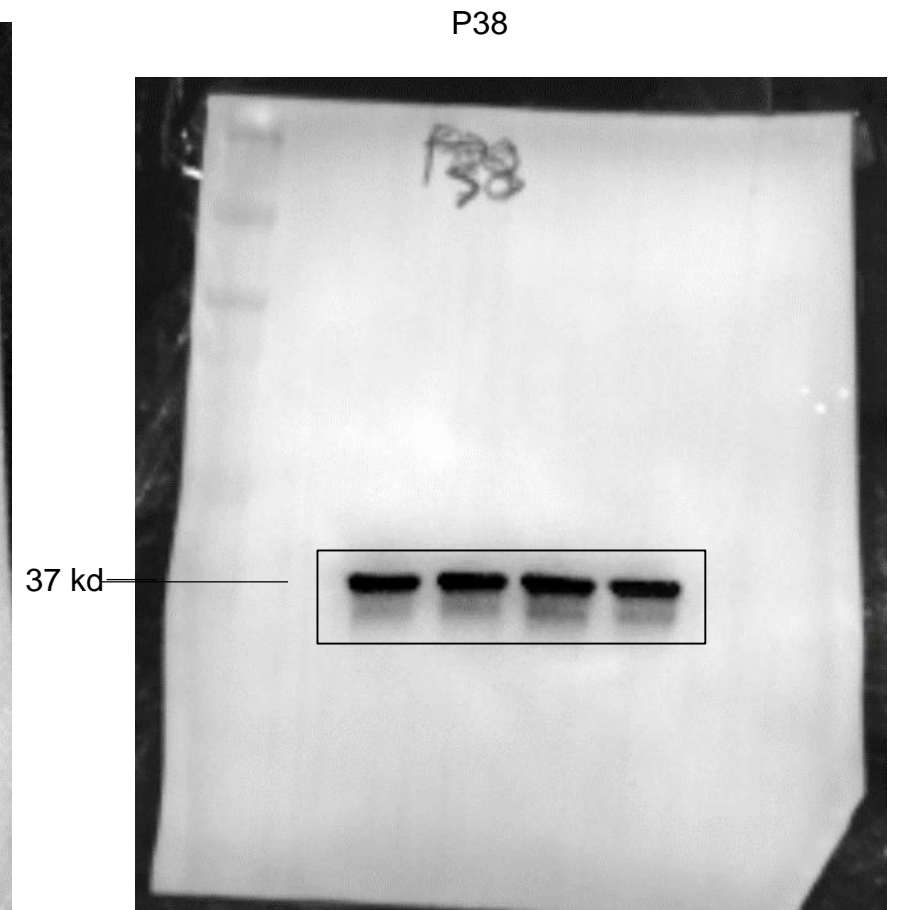

**Fig. 3b**

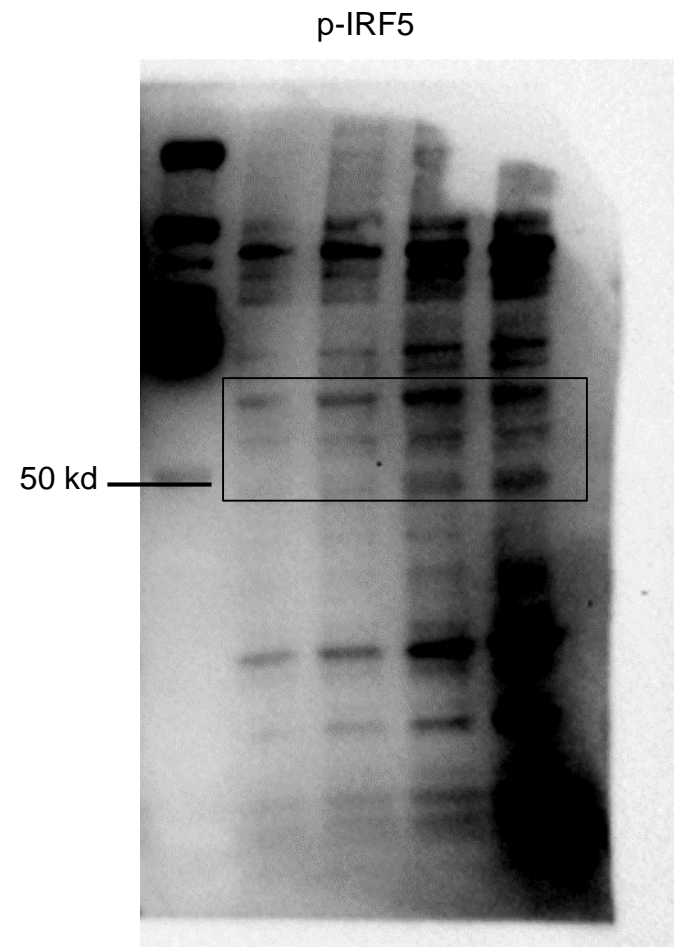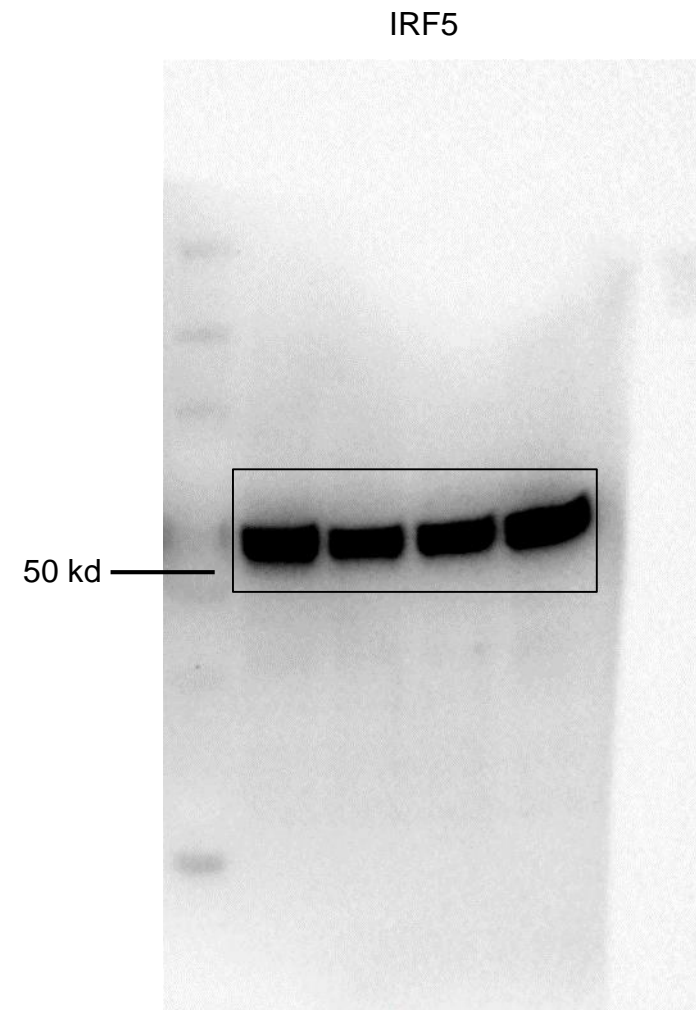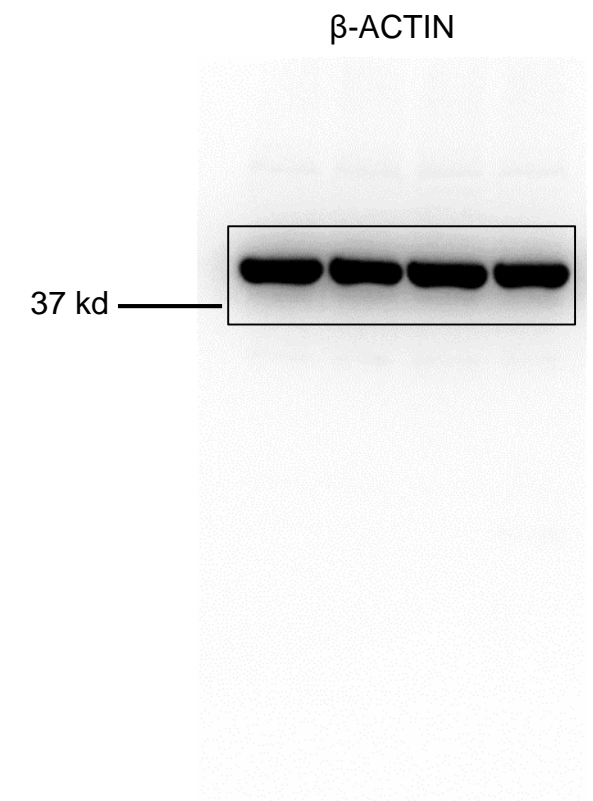

Supplement: Supplementary file 6 — Unprocessed immunoblots. [file 41590_2024_1846_MOESM6_ESM.pdf]

**Fig. 5c**

p-NFκBp65

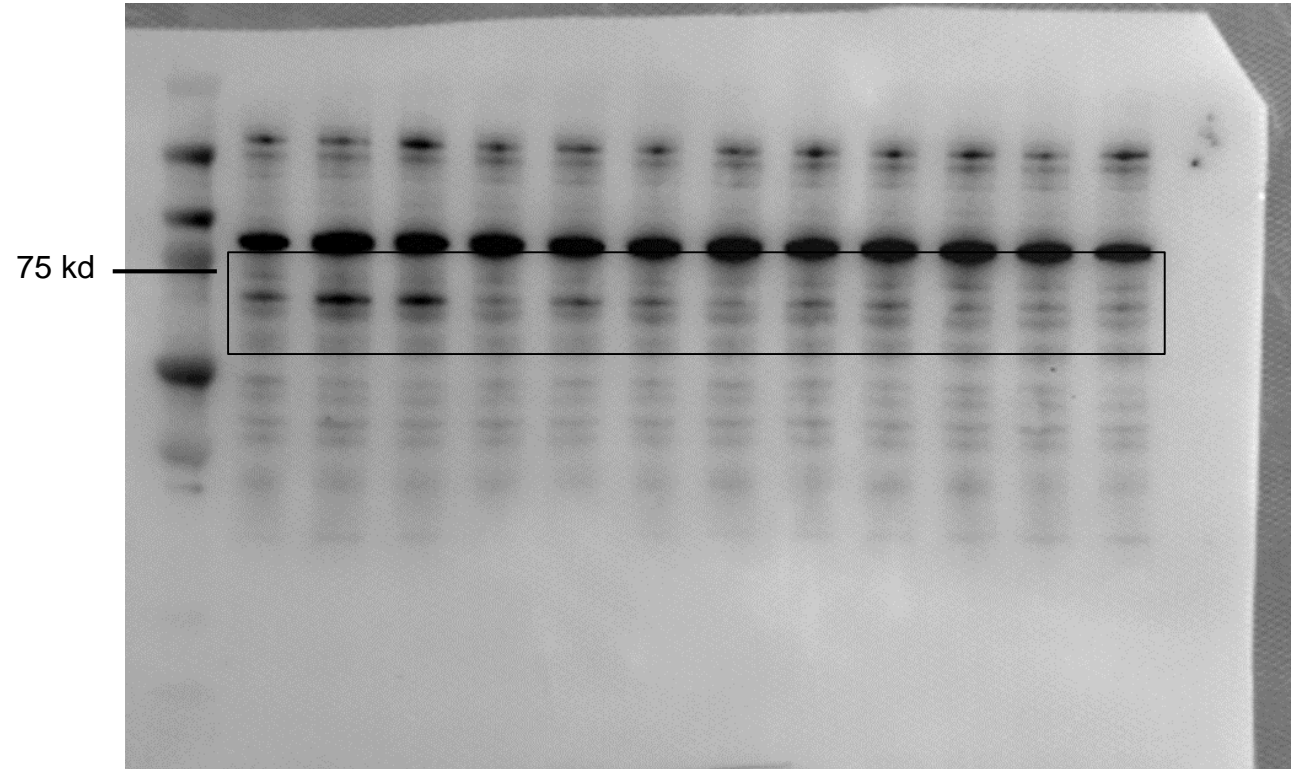

NFκBp65

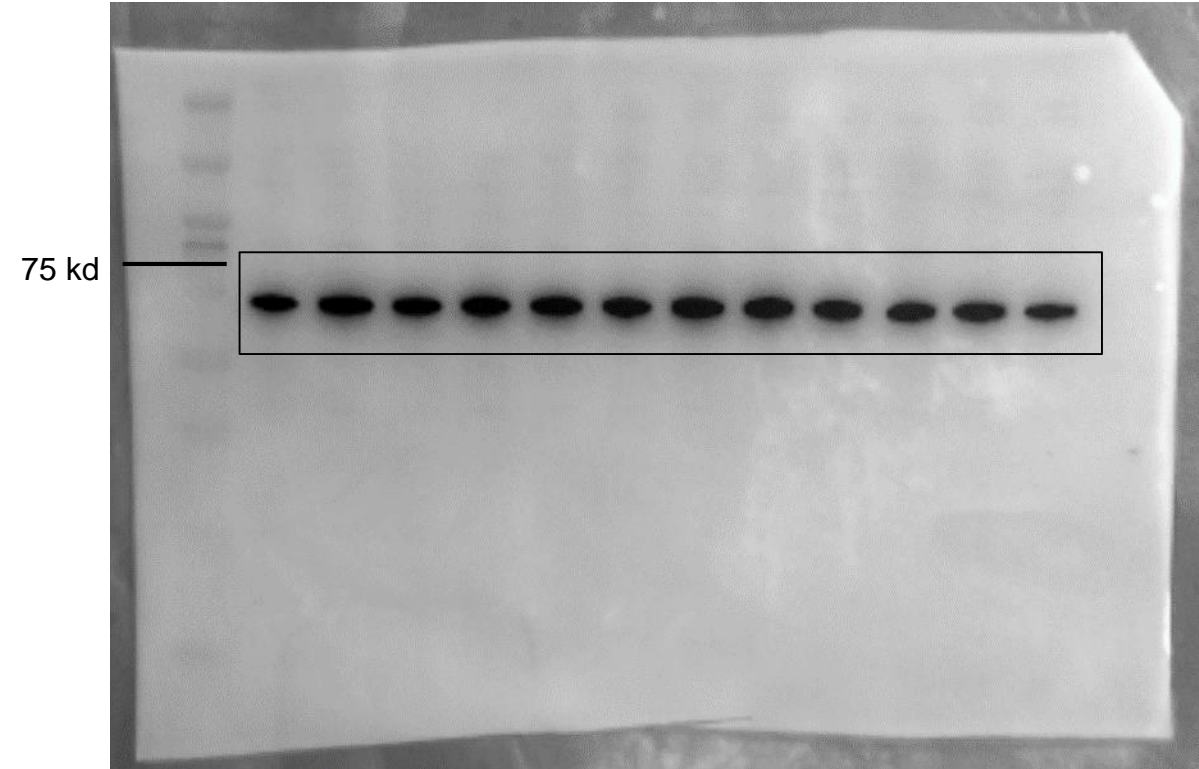

**Fig. 5c**

p-ERK

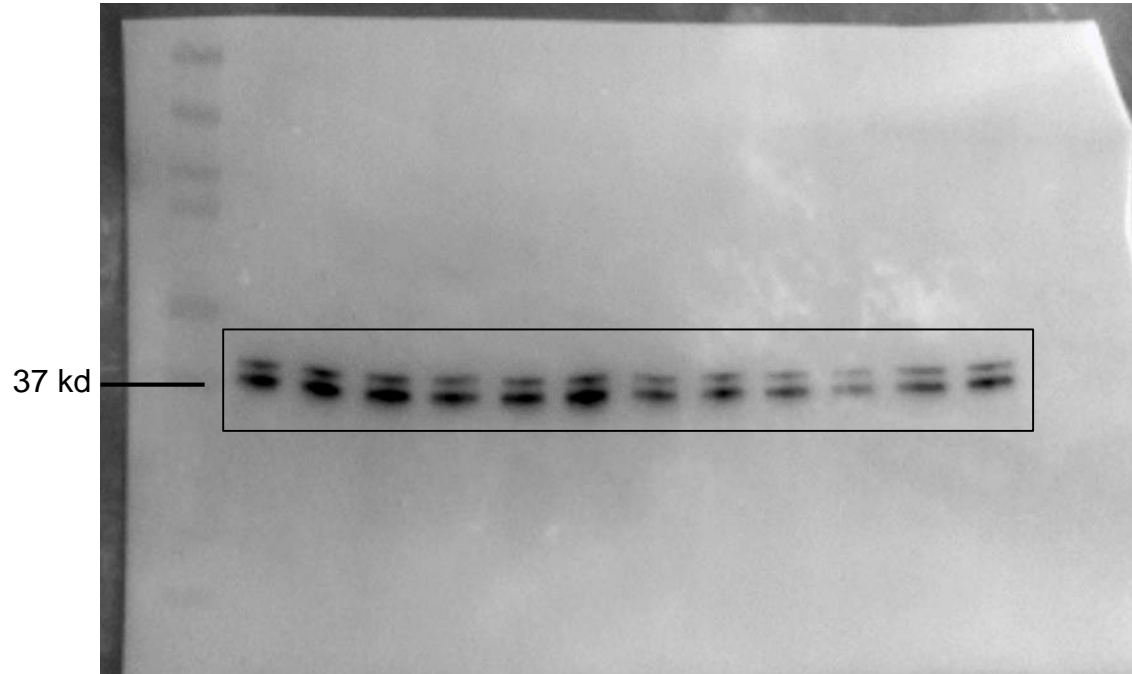

ERK

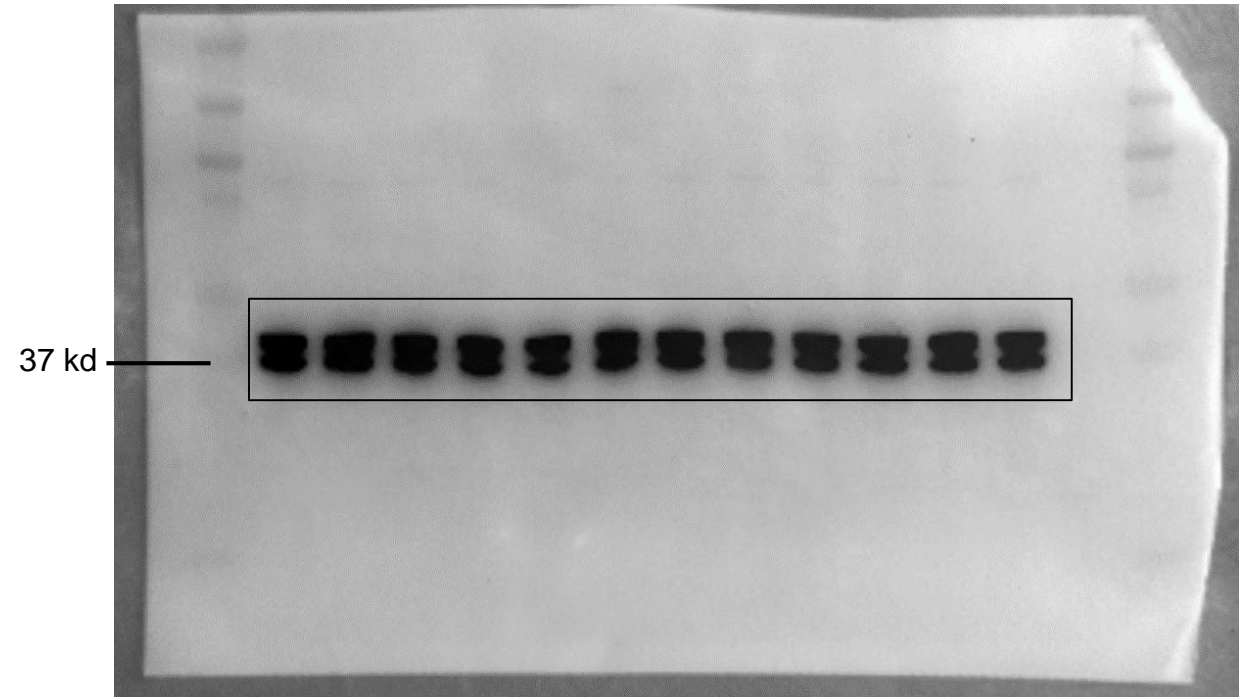

**Fig. 5c**

p-P38

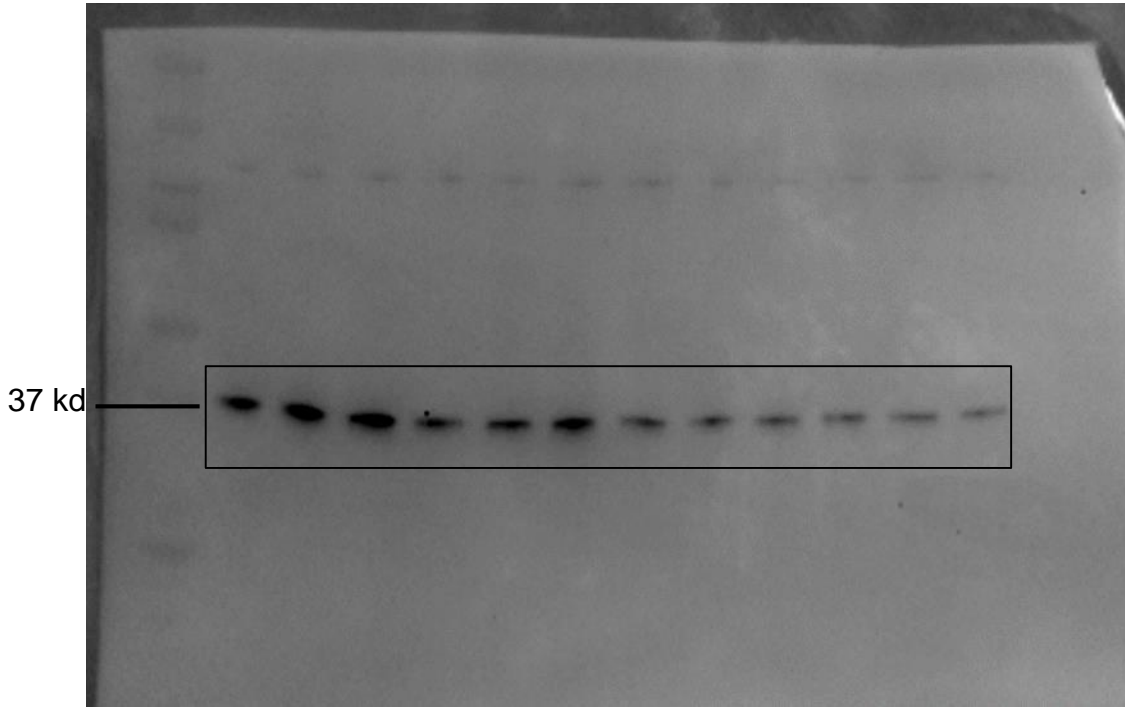

P38

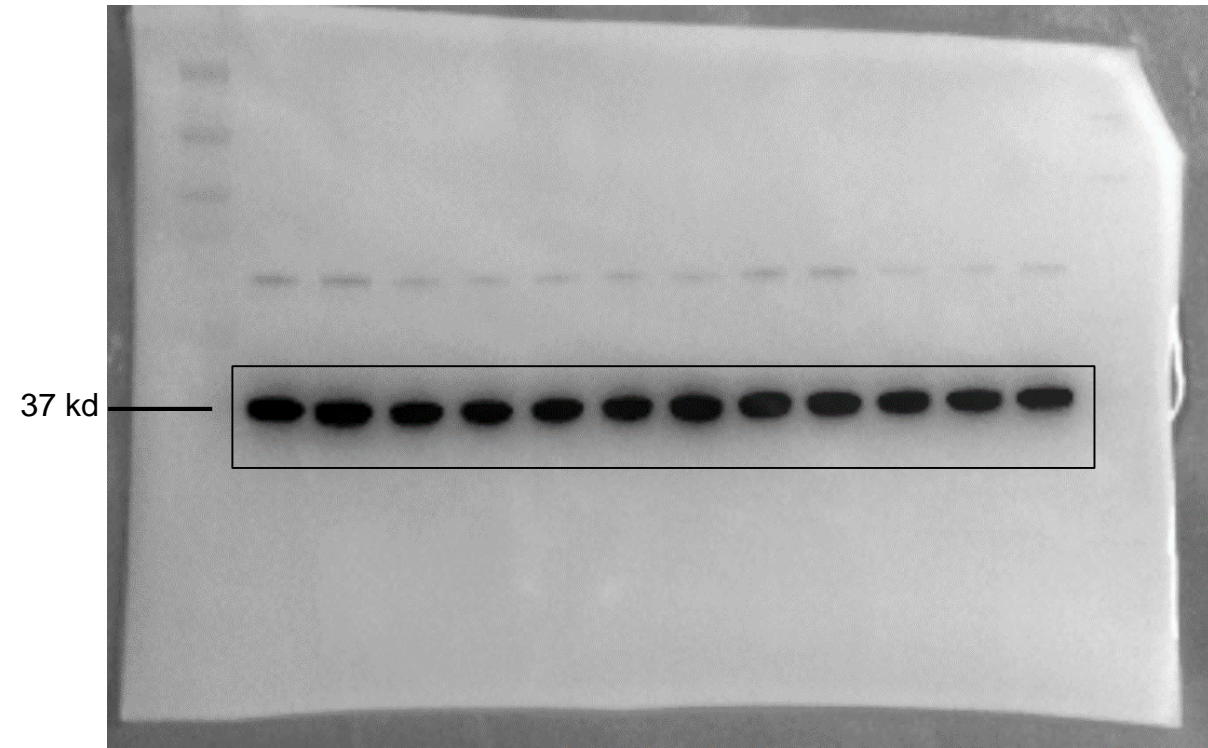

**Fig. 5c**

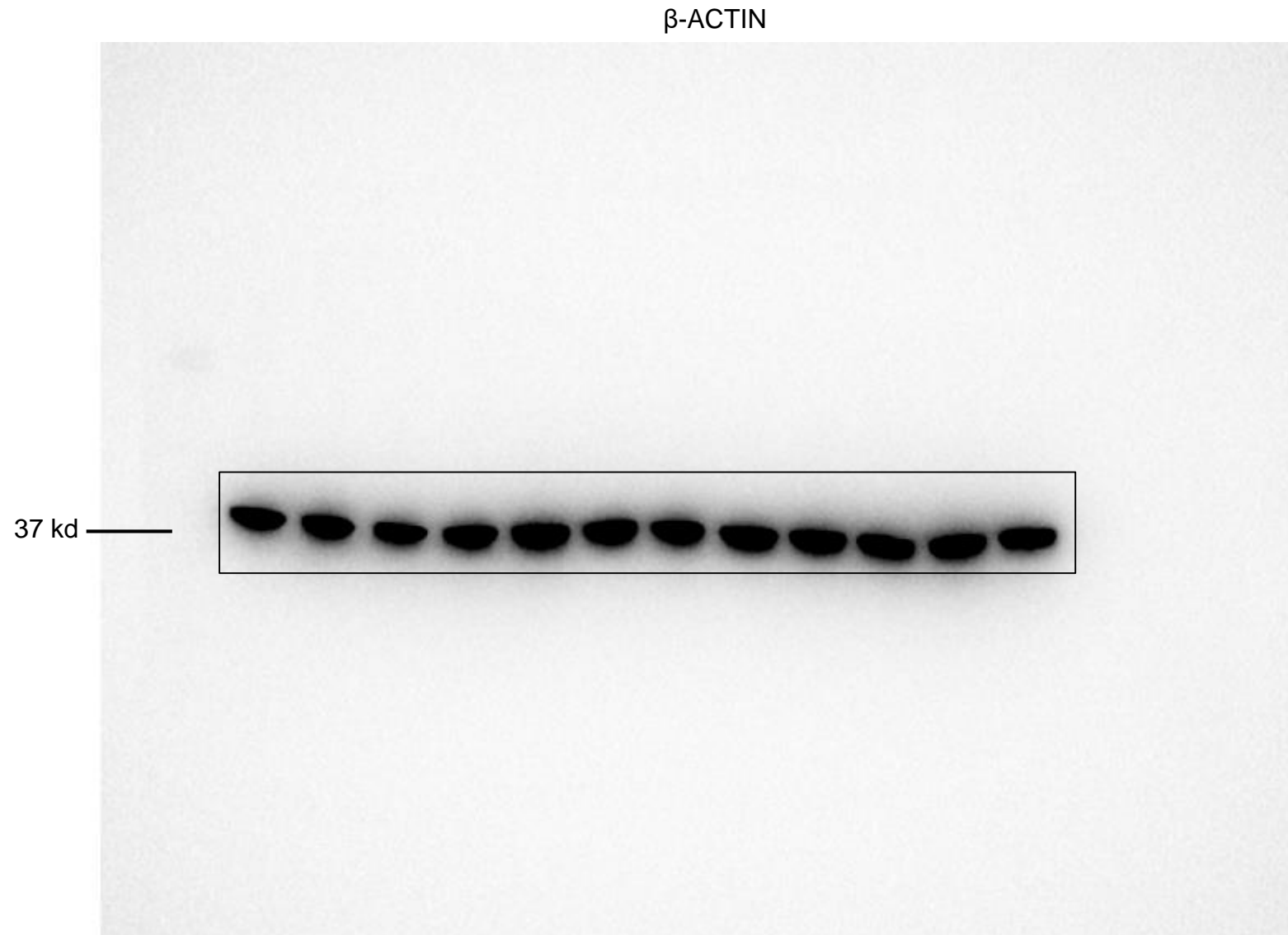

Supplement: Supplementary file 9 — Unprocessed immunoblots. [file 41590_2024_1846_MOESM9_ESM.pdf]

**Fig. 8b**

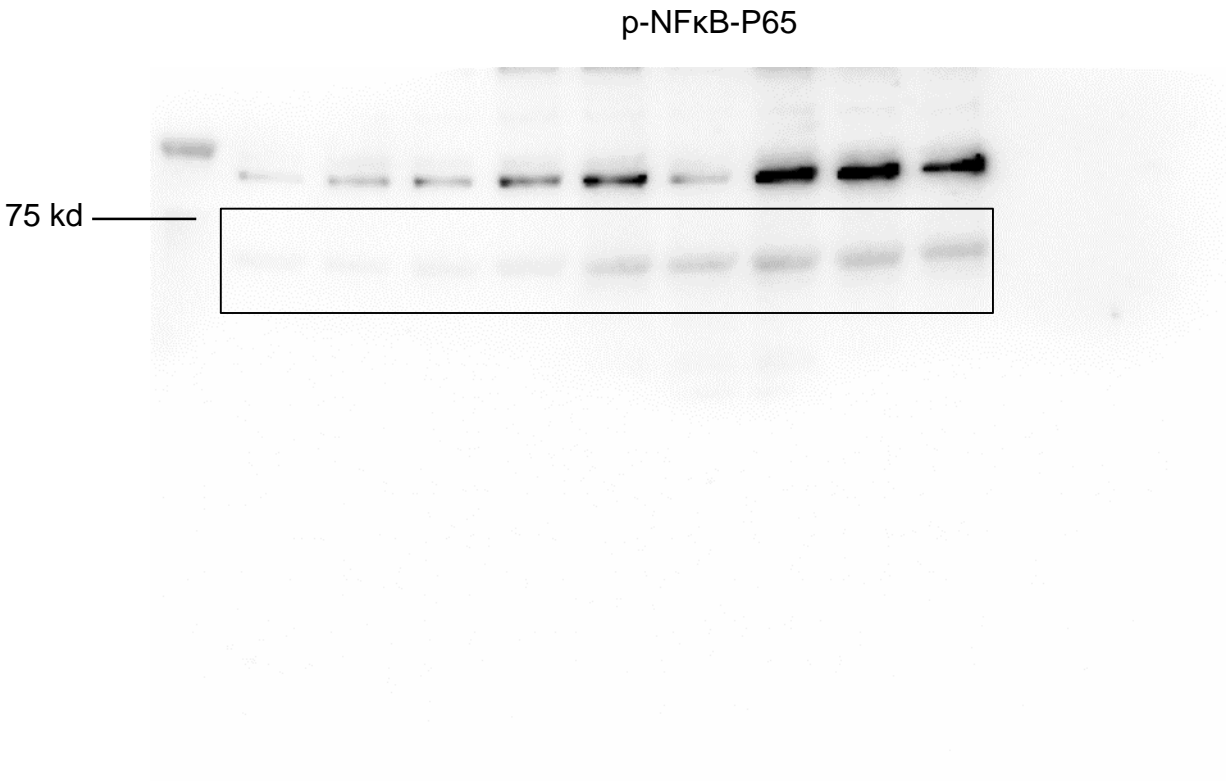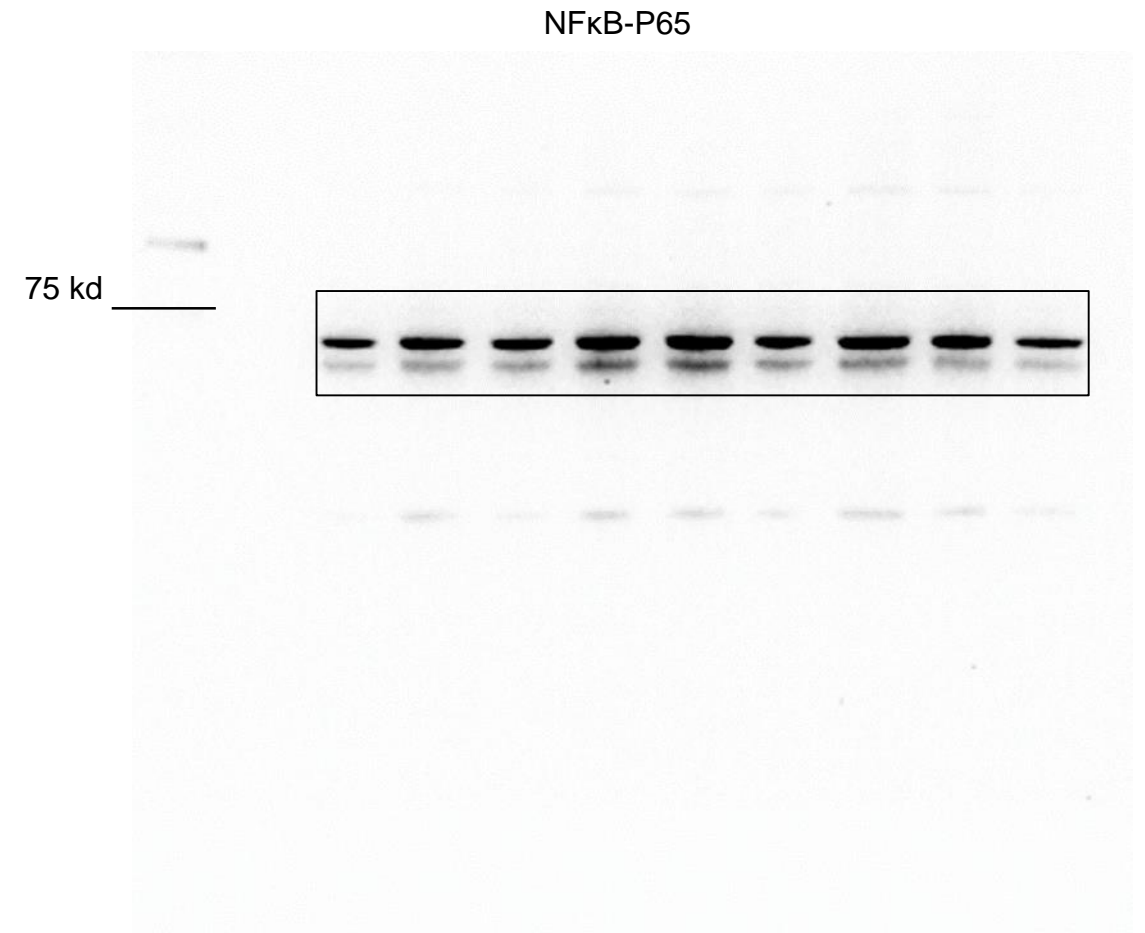

**Fig. 8b**

p-JNK123

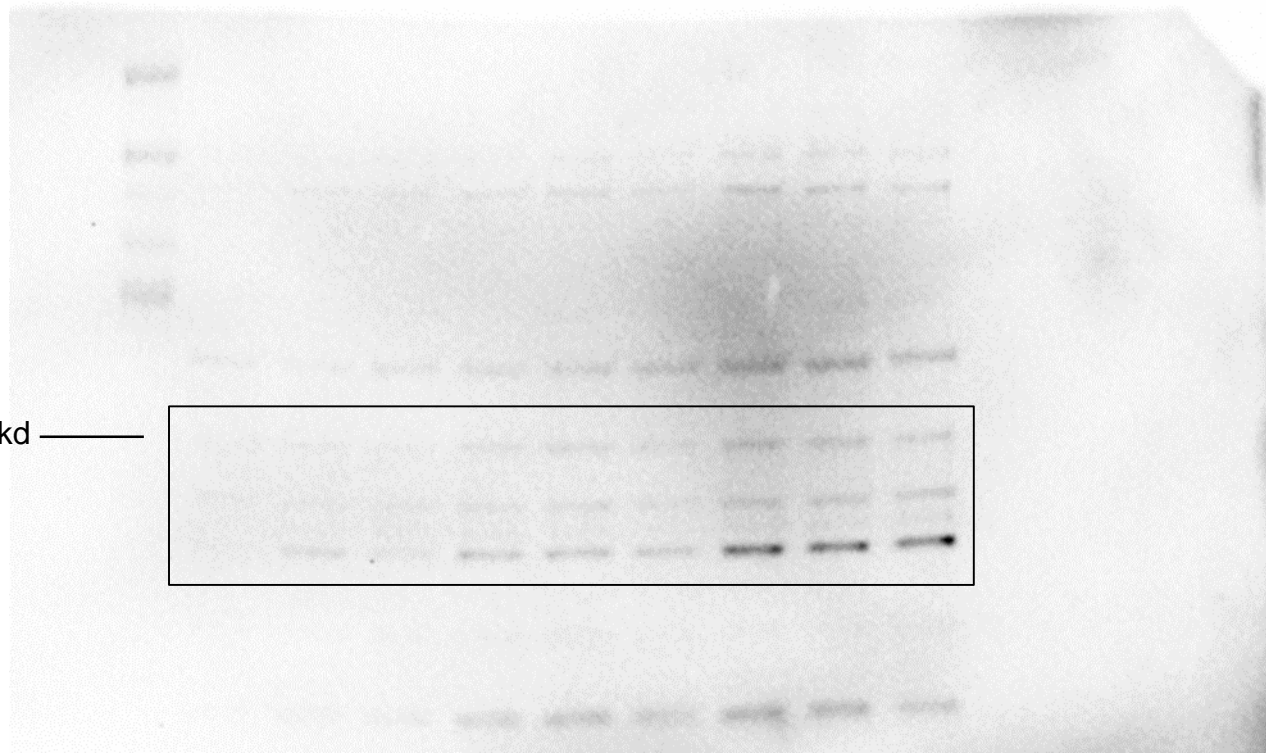

JNK123

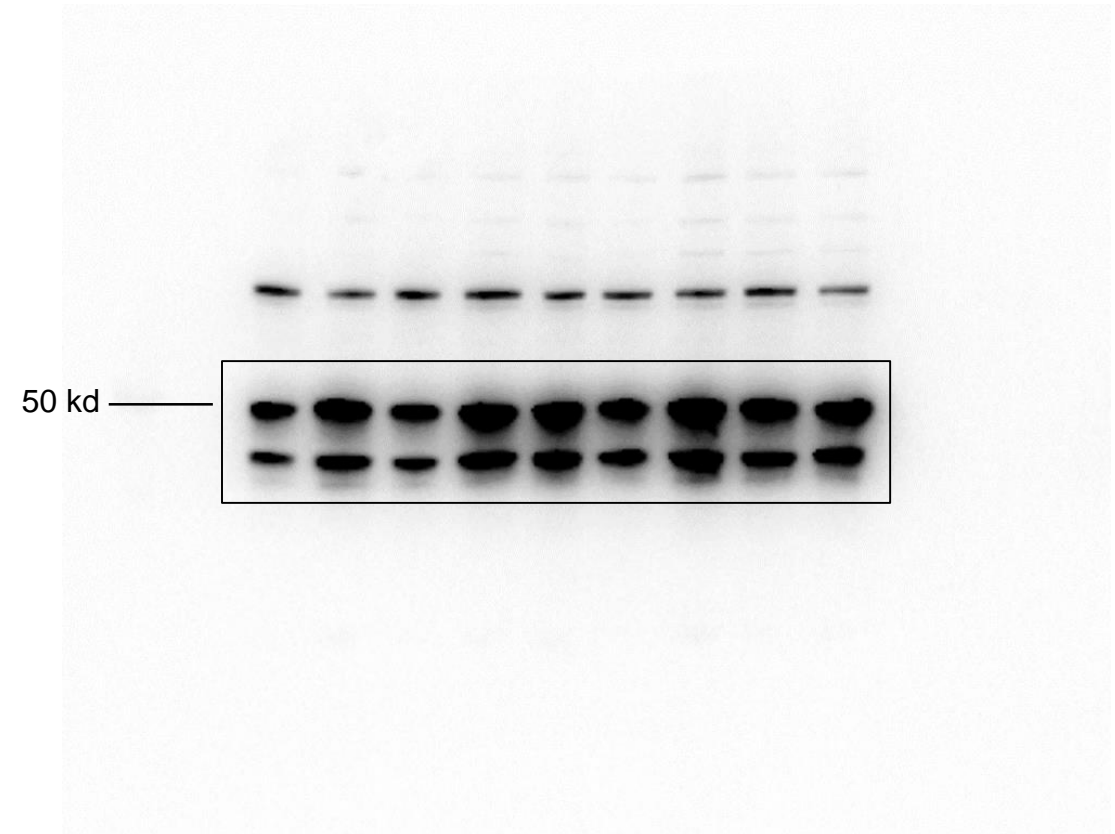

**Fig. 8b**

p-P38

37 kd

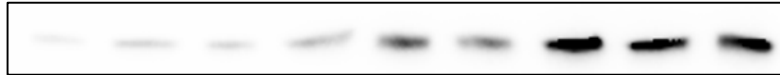

P38

37 kd

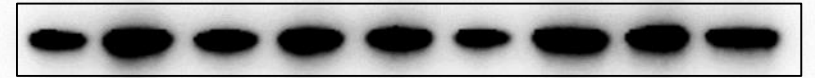

**Fig. 8b**

p-IRF7

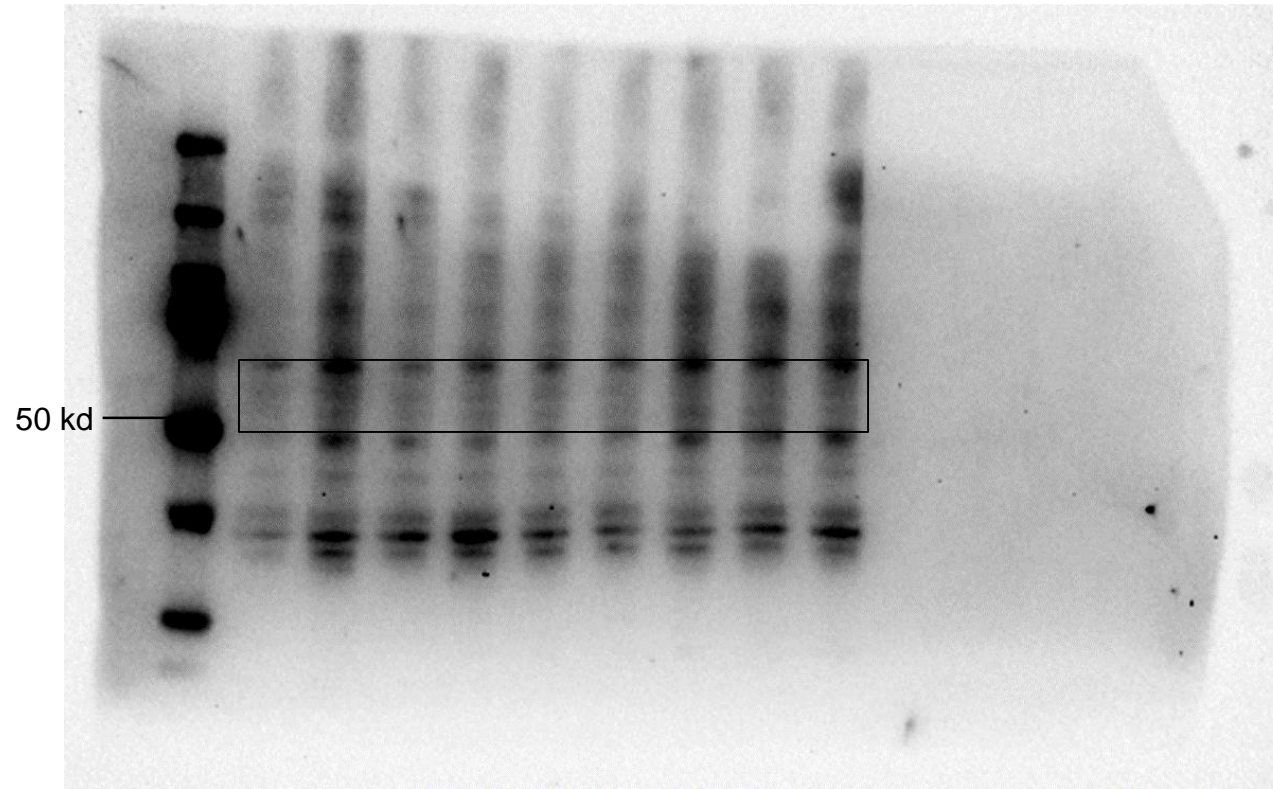

IRF7

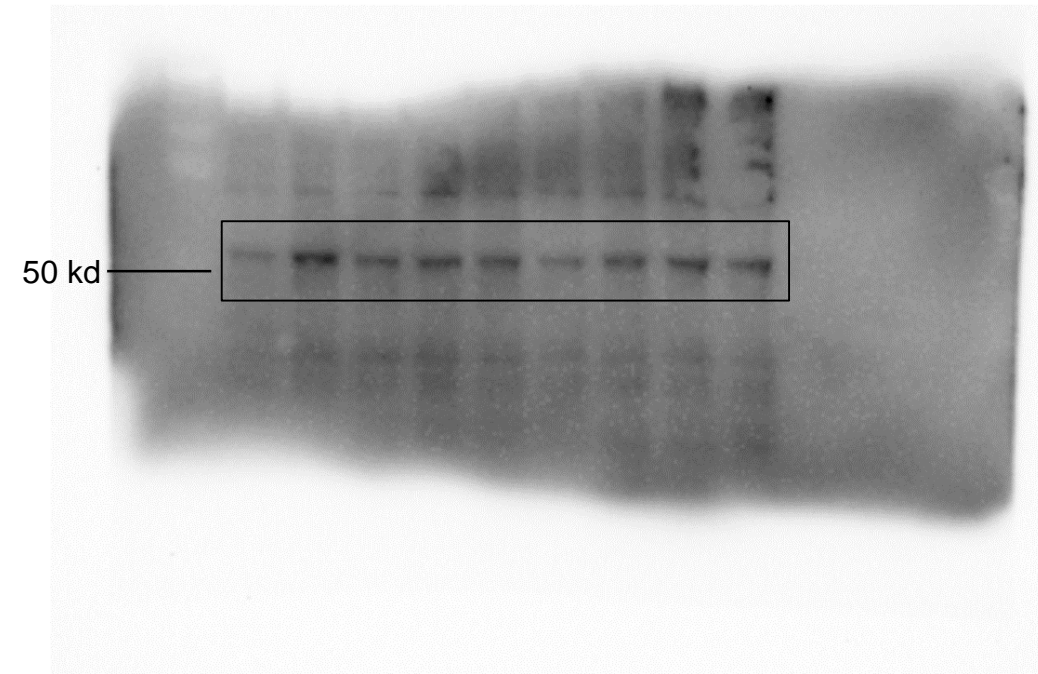

# Fig. 8b

p-IRF5

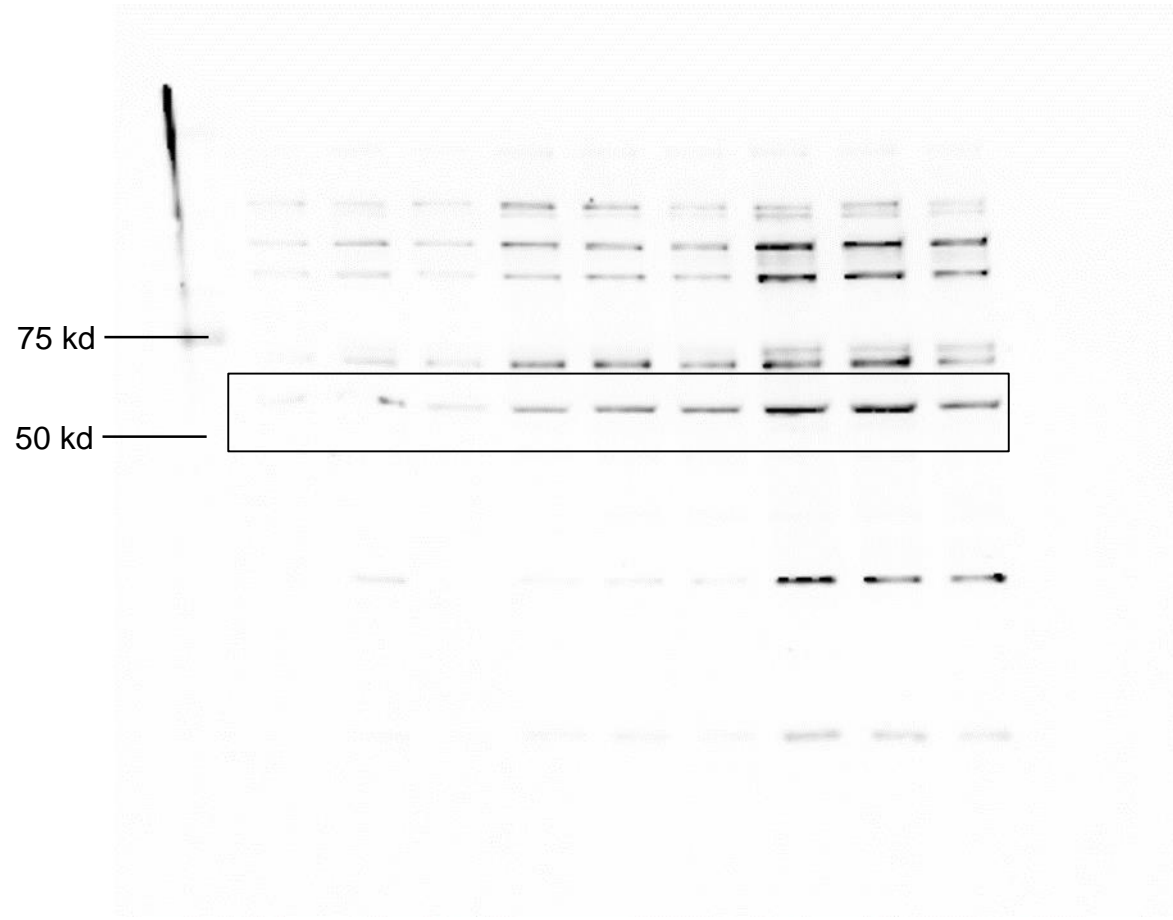

IRF5

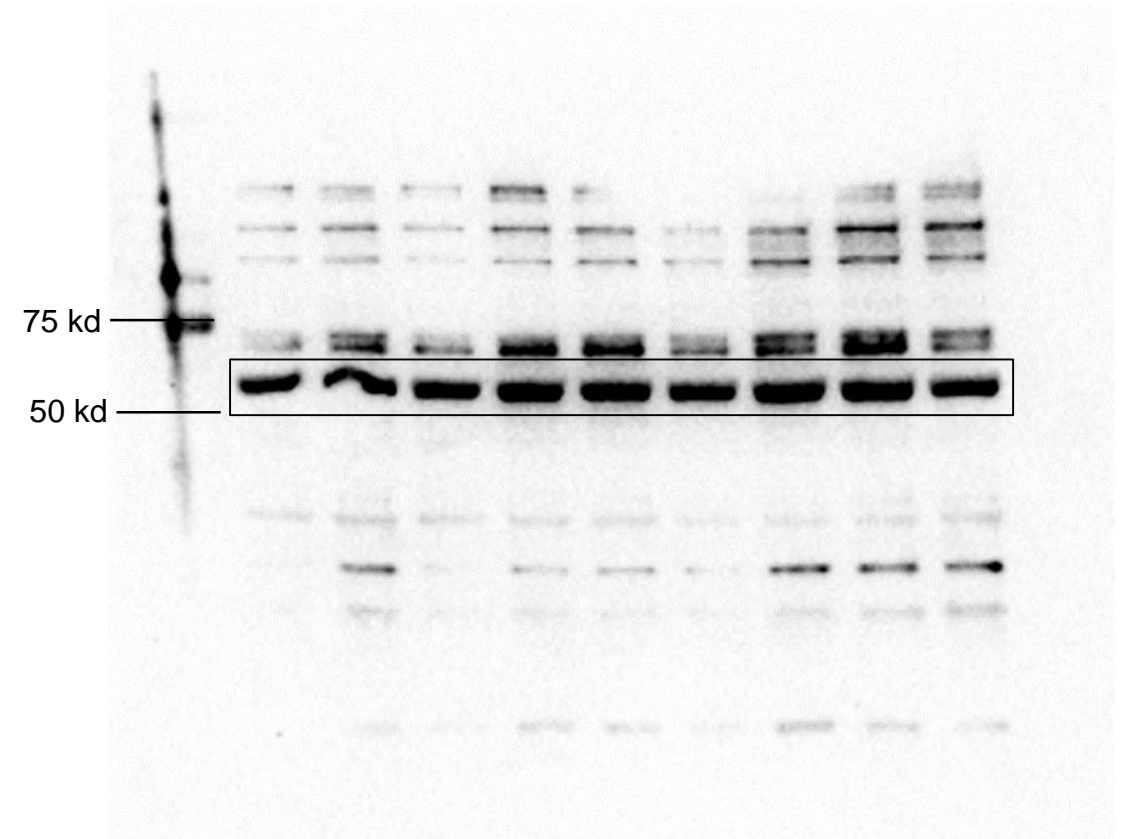

**Fig. 8b**

$\beta$ -ACTIN

37 kd

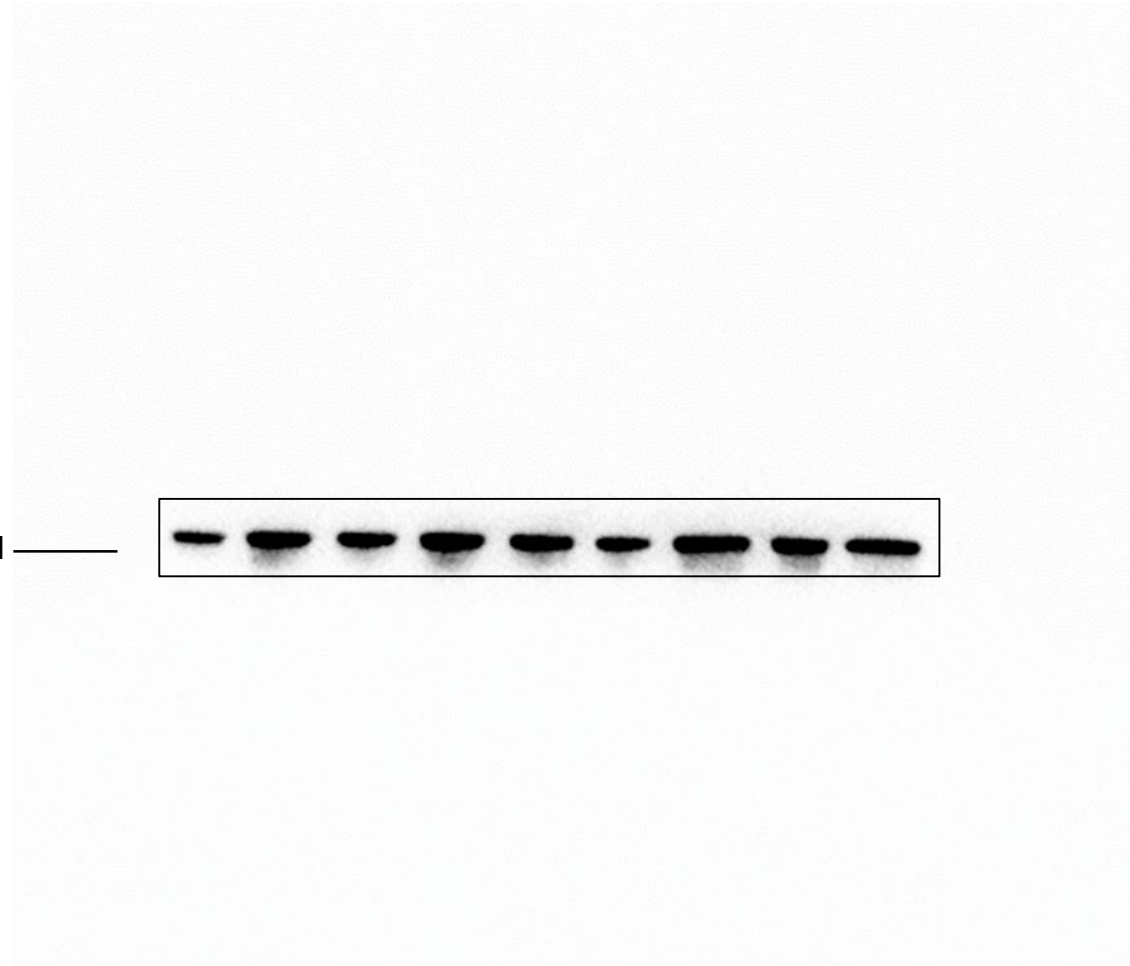

Supplement: Supplementary file 13 — Unprocessed immunoblots. [file 41590_2024_1846_MOESM13_ESM.pdf]

**a**

Negative control

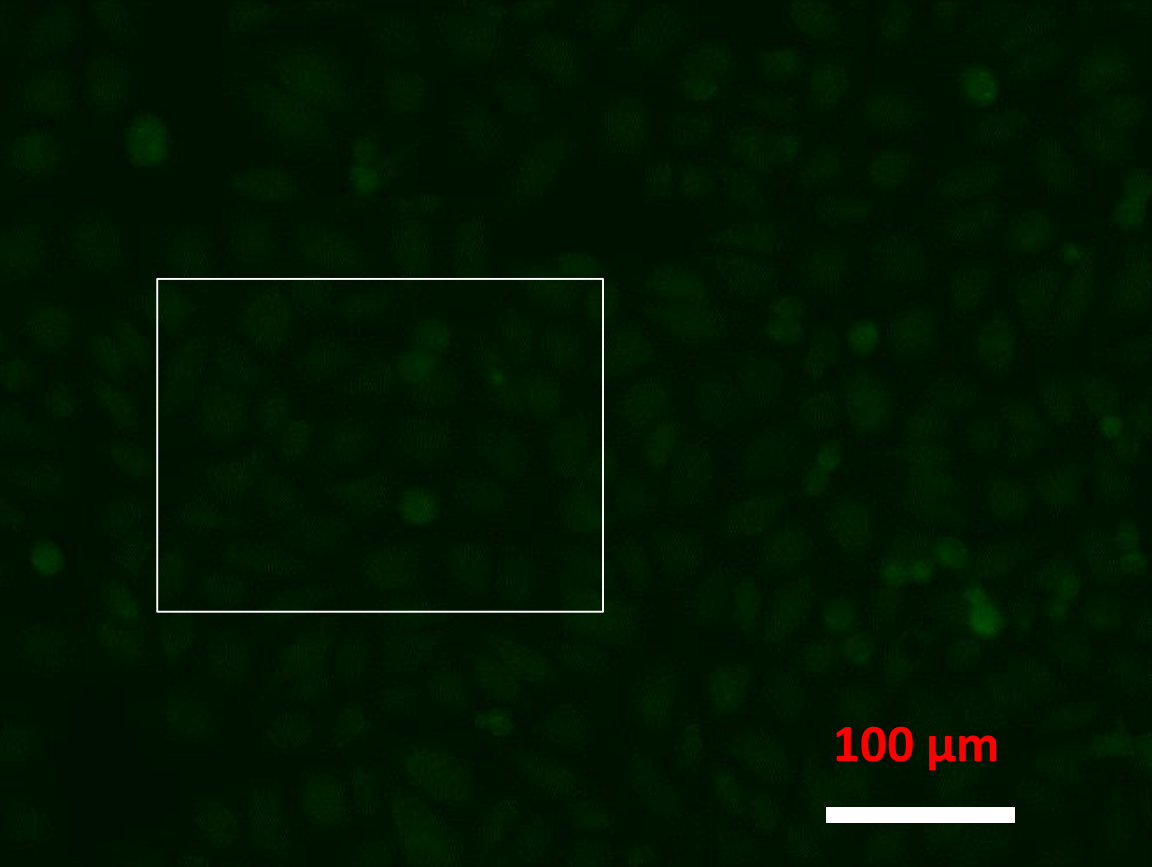

**b**

V117L/SLE P2

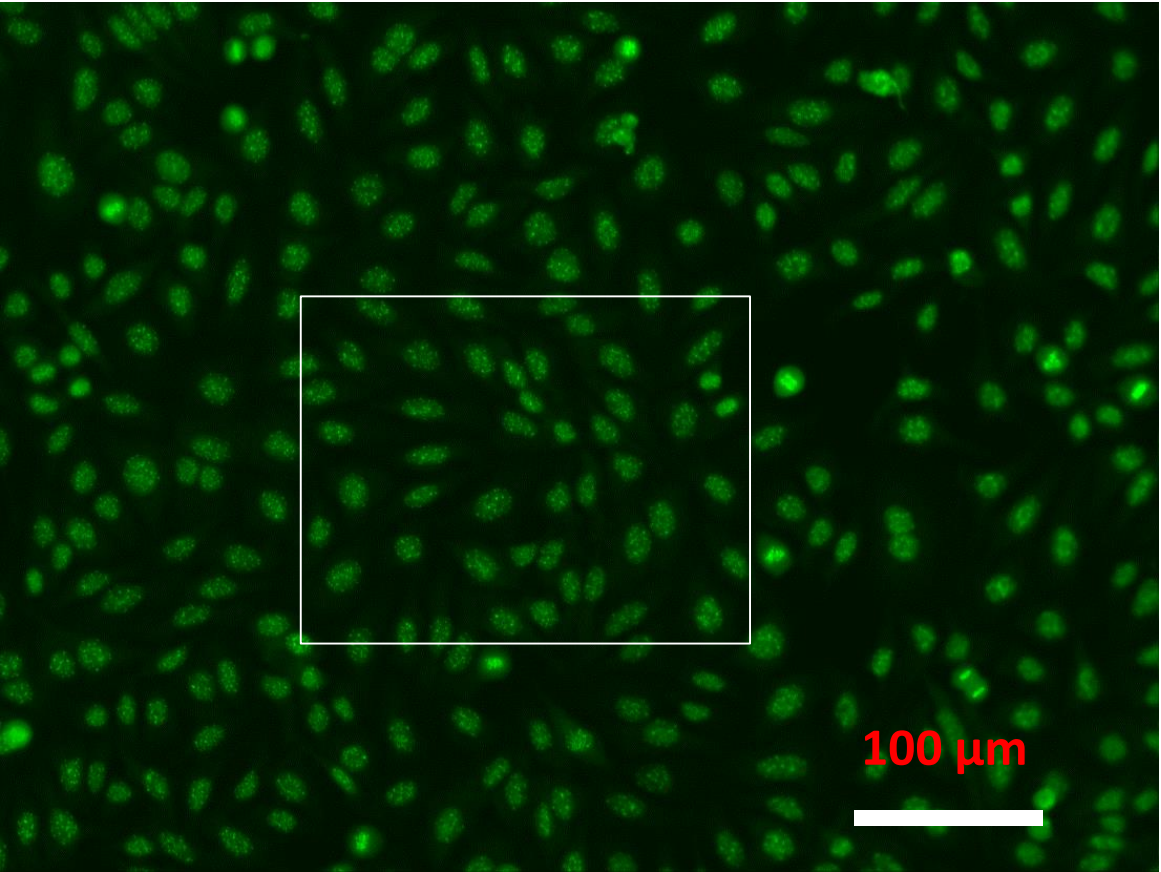

**c**

V117L/SLE P3

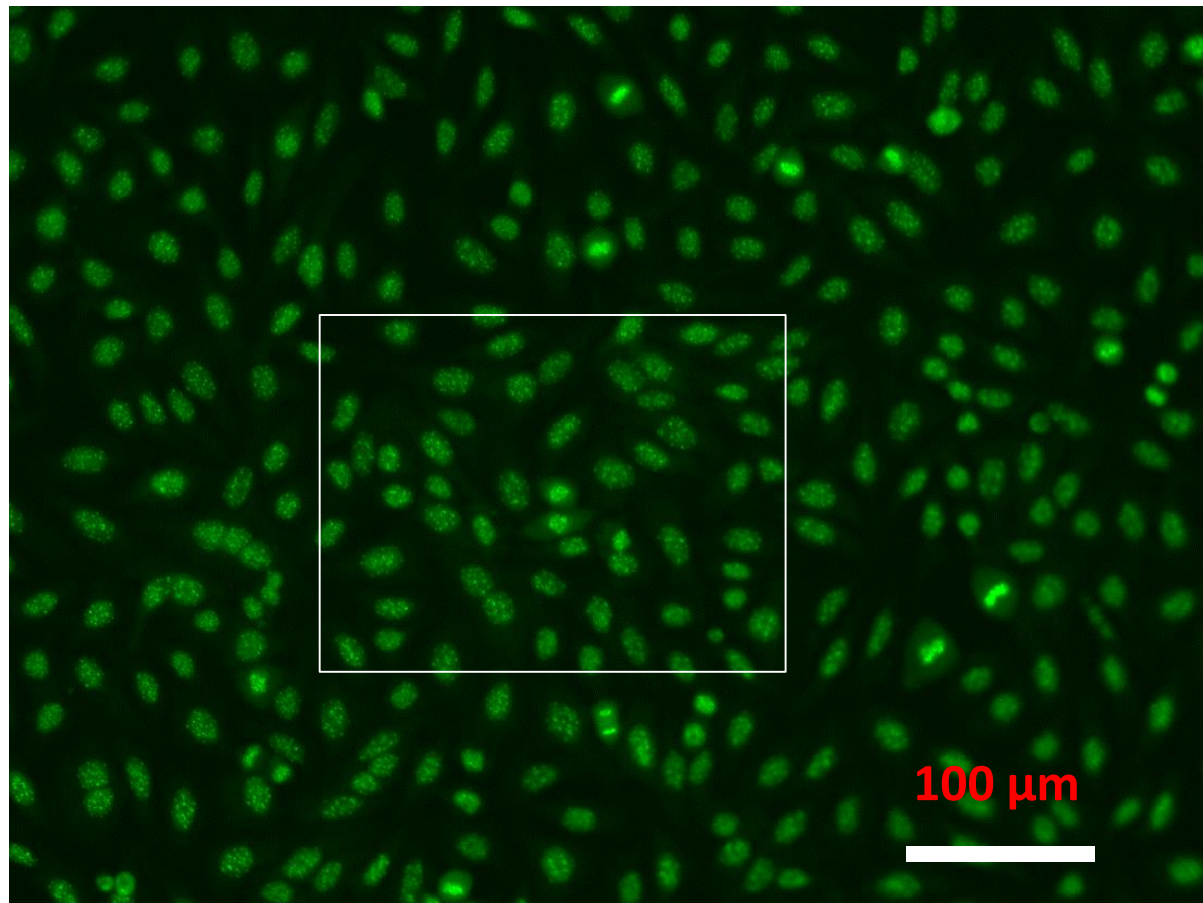**d**

V117L/SLE P4

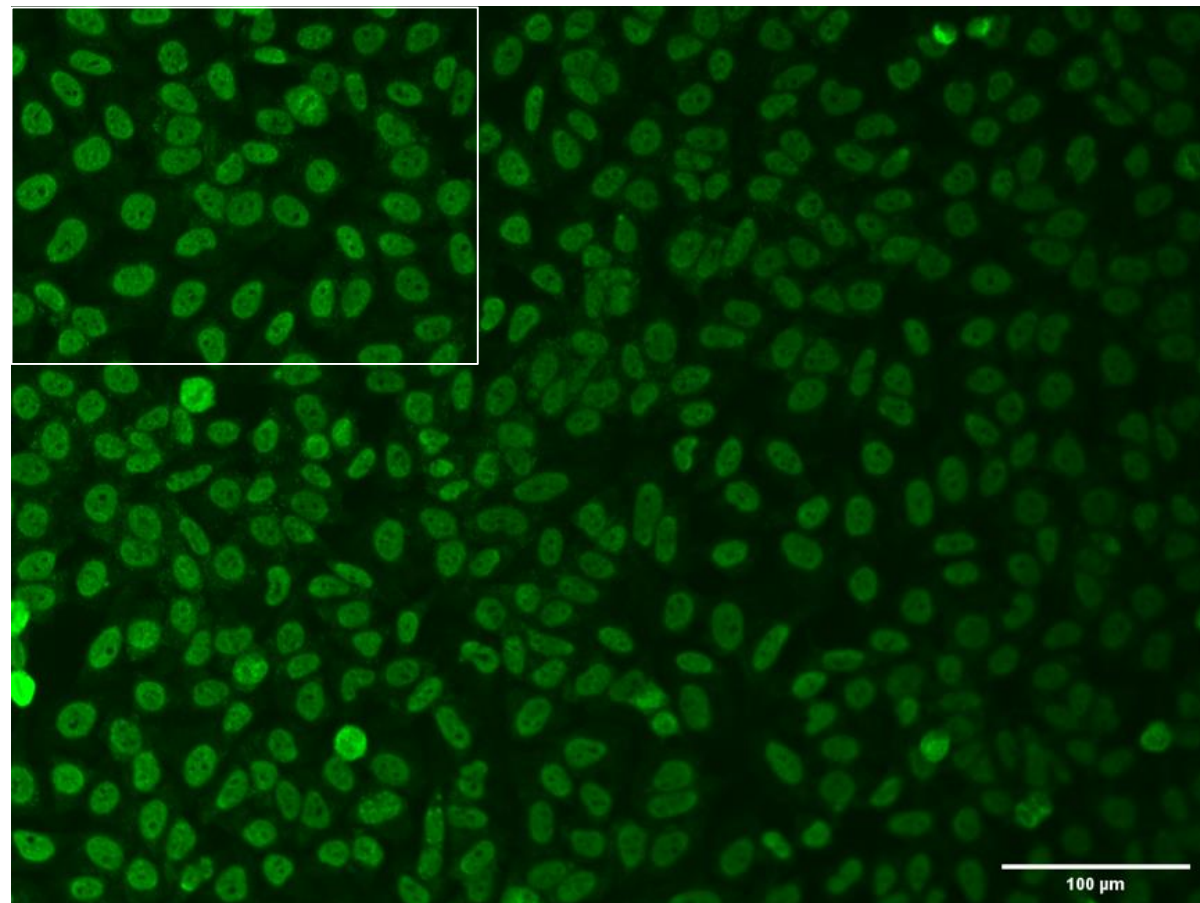

**e**

V117L/SLE P5

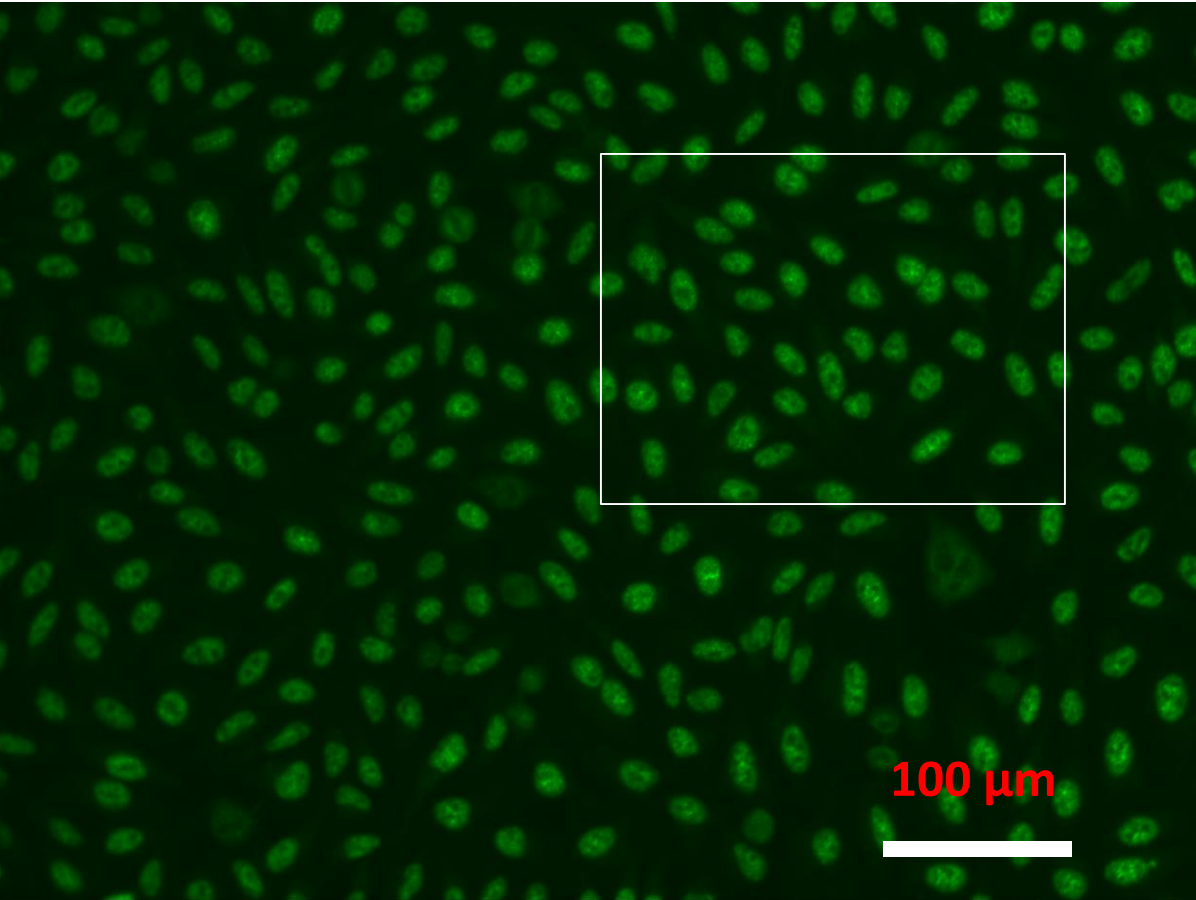

**f**

V117L/SLE P6

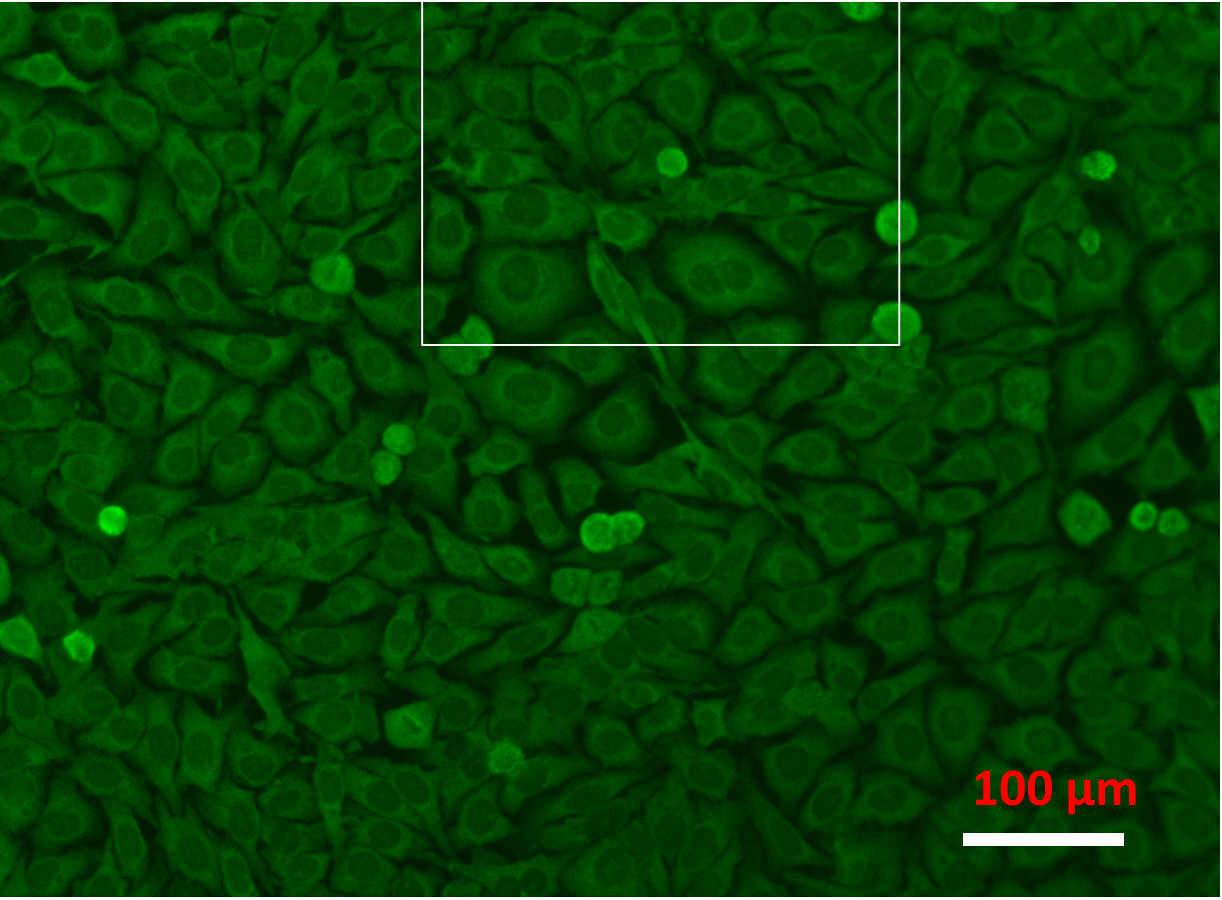

Supplement: Supplementary file 15 — Unprocessed Hep-2 images. [file 41590_2024_1846_MOESM15_ESM.pdf]

# Extended Data Fig. 7a

P-IRAK4

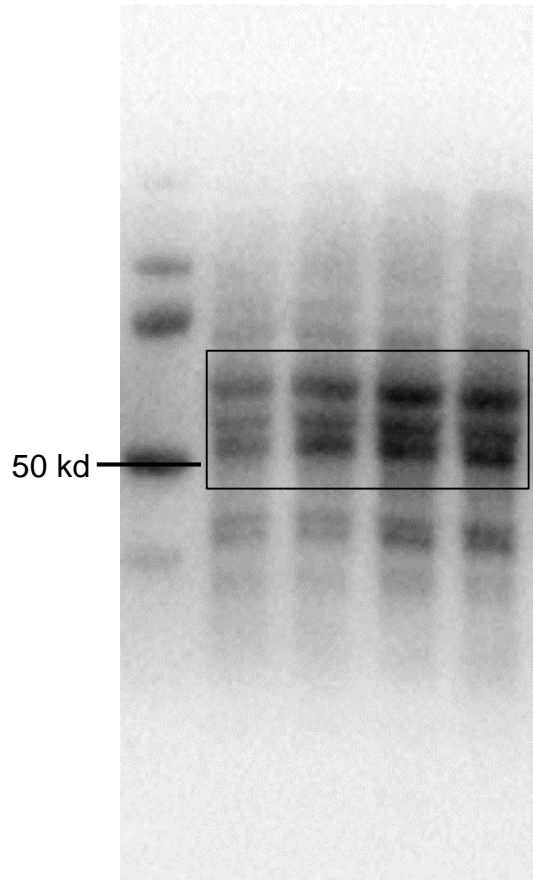

IRAK4

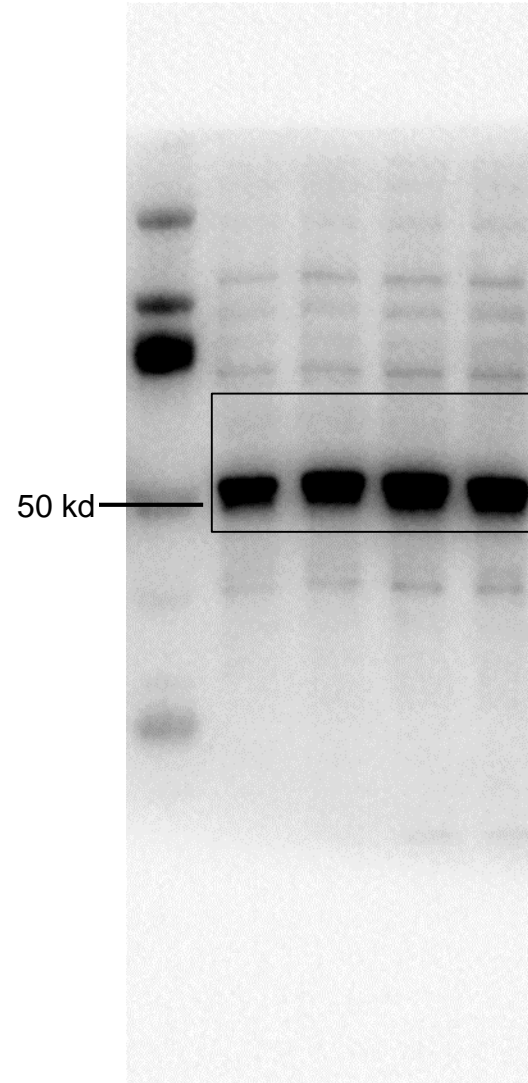

ACTIN

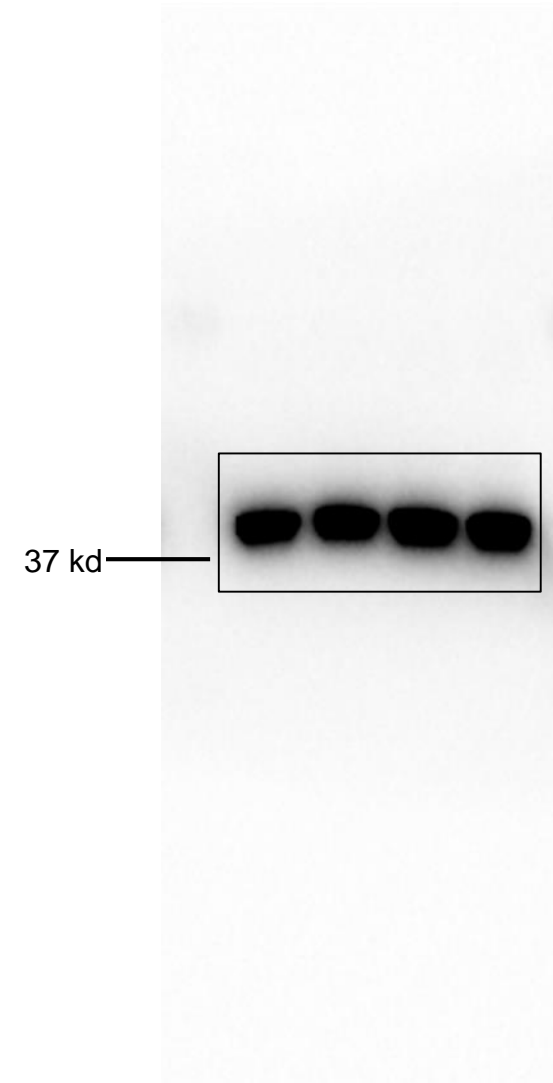

Supplement: Supplementary file 21 — Unprocessed immunoblots. [file 41590_2024_1846_MOESM21_ESM.pdf]
